# Removing array-specific batch effects in GWAS mega-analyses by applying a two-step imputation workflow

**DOI:** 10.1093/bioadv/vbaf317

**Published:** 2025-12-14

**Authors:** Mohammed Kamal Nasr, Eva König, Christian Fuchsberger, Sahar Ghasemi, Uwe Völker, Henry Völzke, Hans J Grabe, Alexander Teumer

**Affiliations:** Department of Psychiatry and Psychotherapy, University Medicine Greifswald, Greifswald 17475, Germany; DZHK (German Centre for Cardiovascular Research), Partner Site Greifswald, Greifswald 17475, Germany; Institute for Biomedicine, Eurac Research, Bolzano 39100, Italy; Institute for Biomedicine, Eurac Research, Bolzano 39100, Italy; Institute of Genetic Epidemiology, Medical Center—University of Freiburg, Faculty of Medicine, University of Freiburg, Freiburg 79106, Germany; DZHK (German Centre for Cardiovascular Research), Partner Site Greifswald, Greifswald 17475, Germany; Interfaculty Institute for Genetics and Functional Genomics, University Medicine Greifswald, Greifswald 17475, Germany; DZHK (German Centre for Cardiovascular Research), Partner Site Greifswald, Greifswald 17475, Germany; Institute for Community Medicine, University Medicine Greifswald, Greifswald 17475, Germany; Department of Psychiatry and Psychotherapy, University Medicine Greifswald, Greifswald 17475, Germany; German Center for Neurodegenerative Diseases (DZNE), Site Rostock/Greifswald, Greifswald 17475, Germany; Department of Psychiatry and Psychotherapy, University Medicine Greifswald, Greifswald 17475, Germany; DZHK (German Centre for Cardiovascular Research), Partner Site Greifswald, Greifswald 17475, Germany

## Abstract

**Summary:**

Combining genetic data from different genotyping arrays (mega-analysis) increases statistical power but introduces array-specific batch effects that may bias results. This project developed a two-step genotype imputation workflow addressing this bias in studies using multiple genotyping platforms.

Genotype data of 10 647 individuals generated using five different arrays were included. The two-step method involved creating intermediate array-type specific panels, which were then imputed against the 1000 Genomes reference panel. Batch effects were assessed using genetic principal component analysis of the combined imputed dataset. Performance was evaluated by comparing imputation quality and allele frequency differences between the two-step and the conventional array-specific imputation. Additionally, concordance with a whole-genome–sequenced subgroup was examined. Genome-wide association analysis on goiter risk and thyroid gland volume was conducted to compare outcomes between both imputation approaches.

The workflow eliminated array-driven batch effect from the first 20 PCs and showed high correlation with the conventional approach for allele frequencies (*r*^2^ > 0.99). GWAS using the two-step imputation confirmed known associations on thyroid traits and revealed novel loci for thyroid volume (*TG, PAX8, IGFBP5, NRG1*), and goiter (*XKR6*), the latter not significant in the conventional imputation.

**Availability and implementation:**

The study provides a workflow for high-quality imputation results without batch effects, fostering genetic analysis involving multiple genotyping arrays.

## 1. Introduction

Genome-wide association studies (GWAS) represent an agnostic approach for identifying genetic associations with common traits and diseases by testing millions of variants with continuous outcomes or between groups. The testing checks allele frequency differences between individuals in a selected population that show different representations of the trait value (Uffelmann *et al.* 2021). GWAS analyses have been utilized in more than 5700 studies, exploring 3300 different traits (Watanabe *et al.* 2019, Uffelmann *et al.* 2021). The results of these analysis enriched our knowledge about disease risk variants, as well as identifying individuals with high disease-risk profiles through risk scores for complex heritable traits (Khera *et al.* 2018, Uffelmann *et al.* 2021).

The power to detect associations increases with sample size and number of variants tested (Willer *et al.* 2010, Khera *et al.* 2018), rare and low frequency single nucleotide variants (SNVs) are of high interest. This requires the inclusion of large sample sizes in the experimental design, which is complicated or even unrealistic for rare diseases and low number of cases in the investigated populations. One alternative approach is a meta-analysis of summary statistics from different GWAS analyses of the same trait, performed either on same or different populations (Willer *et al.* 2010, Mägi *et al.* 2017). Another approach is to combine the individual-level data of samples from different cohorts to perform a mega-analysis (Abou-Khalil *et al.* 2018).

Meanwhile, genetic imputation is a reliable method to estimate alleles of variants not directly genotyped on an array. Based on linkage disequilibrium (LD), genetic imputation can significantly increase coverage of the human genome when using different commercial genotyping arrays (Burton *et al.* 2007, De Marino *et al.* 2022). This method is utilized as an alternative for expensive whole genome sequencing (Quick *et al.* 2020).

GWAS on imputed variants and subsequent meta-analysis are frequently utilized cost-effective approaches for conducting large GWAS (Pasaniuc *et al.* 2012), however, they are also associated with technical limitations (Palmer and Pe’er 2016). If the sample size or number of cases in a specific cohort especially in a (nested) case-control design is low, the analysis results are less reliable because the effective number of samples is too small for the association models like linear regression, logistic regression, or mixed-effects models, particularly when it comes to low-frequency variants. In addition, the analysis workload substantially increases with the number of cohorts included in a project (Winkler *et al.* 2014). Former studies have shown that both meta-analyses and mega-analyses using individual participant data are mathematically equivalent (Lin and Zeng 2010), and also comparable when using imputed genotypes (Gorski *et al.* 2019). However, previous studies have mainly focused on data from cohorts genotyped on the same array type. Highlighting challenges in mega-analysis, where individual-level data from multiple cohorts genotyped on different arrays are combined.

We identified a concerning technical bias when combining the imputed data of individuals from genetically homogeneous North-Eastern German cohorts that were genotyped using diverse array types (Völzke *et al.* 2011, Grabe *et al.* 2014). Principal component analysis (PCA) conducted on the quality-controlled genotype data revealed variation influenced by the array type. Notably, this variation was detected upon both imputation against the haplotype reference consortium (HRC) (McCarthy *et al.* 2016) and the 1000 Genomes v5 (1000G) reference panels (Auton et al. 2015). This batch effect was persistent regardless of using genotype dosage or best-guess genotype for the PCA.

Here, we propose a newly developed workflow, to minimize the technical bias due to batch effect when combining genotype data from different arrays. The workflow is composed of two imputation steps. First we impute the included genotype datasets of each array type pairwise against each other and then create a panel of overlapping variants for each imputation outcome. Finally, we impute the generated intermediate panel against one of the commonly available large panels. We evaluate the outcome of the new workflow in comparison to conventional imputation approaches. In an application example, we conducted a GWAS analysis on thyroid gland volume and goiter, identifying new associations with goiter while demonstrating the robustness of our imputation workflow by validating known associations.

## 2. Methods

### 2.1 Workflow design overview

**Figure 1.**
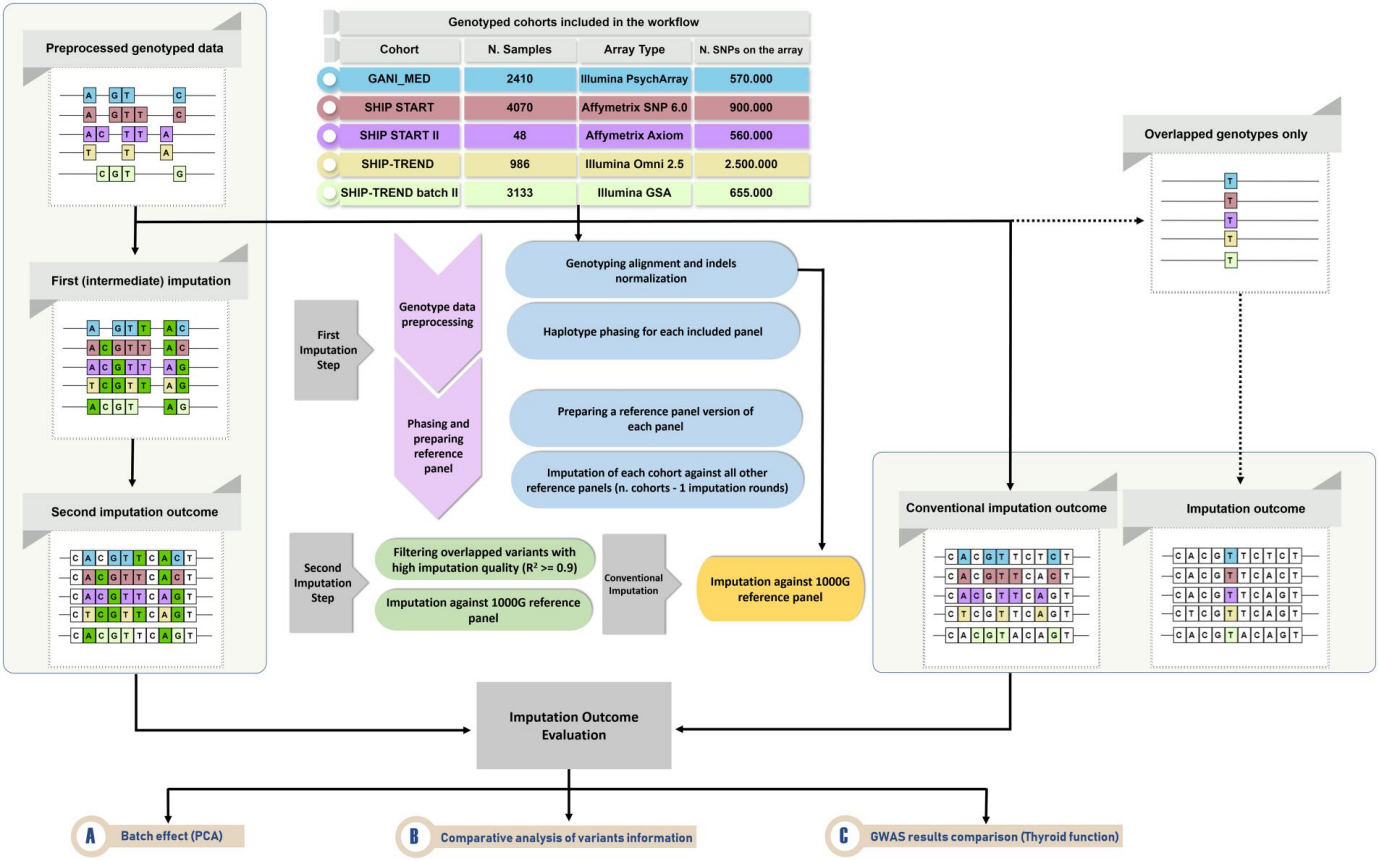
Overview of the designed workflow. The light grey box on the left illustrates the two-step imputation, and the box on the right the conventional (single-step) imputation per array using all variants and their intersection, respectively. Variants are colored by their array source, where variants colored in dark green represent the variants imputed in the intermediate imputation phase (first imputation), while variants colored in white are imputed from the general (i.e. 1000 Genomes) reference panel in both the two-step and conventional imputation. The workflow diagram in the center provides details of each imputation step, and lists the evaluation steps of the imputation results.

In this project, three different approaches for genetic imputation have been conducted. The first proposed approach is a new two-step imputation process. The second and third approaches are conventional single-step imputation for comparison with the newly proposed method. We modified the third approach to be a single-step imputation using only the intersecting variants genotyped on all included array types. The genotype data that were used for imputation were the same in both imputation approaches. We used data from five different arrays (Affymetrix SNP 6.0, Affymetrix Axiom, Illumina Omni 2.5, Illumina GSA, and Illumina PsychArray) obtained from samples of the German GANI_MED (*n = *2410), SHIP-START (*n = *4070), and SHIP-TREND (*n = *4119) cohorts (Fig. 1). The individuals genotyped on the Affymetrix Axiom array (*n = *48) were a subset of the Affymetrix SNP 6.0 samples, whereas all other individuals were genotyped only once. Detailed information regarding the included datasets, sample sizes, and genotype arrays are provided in Fig. 1 and the Methods, available as supplementary data at *Bioinformatics Advances* online. For evaluation of the imputation performance, a whole-genome sequenced subset of 192 individuals from SHIP-TREND cohort has been used for genotype matching concordance checking.

### 2.2 Data pre-processing and phasing

Genotype quality control was performed using PLINK (Chang *et al.* 2015) as reported previously (Sterenborg *et al.* 2024, Teumer *et al.* 2016). Briefly, arrays with genotype call rate <94%, as well as variants with missing call rate >5%, Hardy-Weinberg equilibrium *P* value <10^−4^, and monomorphic SNVs and singletons were excluded. All the included genotype datasets were aligned to reference genome build (GRCh37) using BCFtools software followed by retaining only sites with at least one alternative allele (Danecek *et al.* 2021). Haplotype phasing preceding the first imputation round was performed for each genotype dataset using Eagle2 (5 phasing iterations and number of conditioning haplotypes = 10^4^) and estimated to hg19 mapping (Loh *et al.* 2016). We did not use external reference panel for phasing to keep population diversity information.

### 2.3 Two-step imputation

In the first step, the intermediate imputation, each phased panel had a second version to be used as a reference panel by compressing the phased reference panel files with minimac4 imputation software (Das *et al.* 2016). Each of the five pre-processed genotype arrays was imputed pairwise against the other four, yielding 20 imputed datasets. Using an R scripted algorithm with VCFtools and BCFtools for variants filtration (Danecek *et al.* 2021), each of these imputed variant sets had being subsetted to the variants overlapping between all generated panels with high imputation quality (*R*^2^ ≥ 0.9), selecting the source panel with the highest imputation quality score for each variant. The outcome of the first-step imputation was one VCF file for each cohort (i.e. array type) dataset, which would be used as input for the second imputation.

In the second imputation step, the generated intermediate genotype datasets were used as input for the second imputation against 1000G reference panel using the Michigan imputation server (Das *et al.* 2016). Eagle2 was selected for phasing without (additional) imputation quality filters applied at this stage of the imputation.

### 2.4 Conventional imputation

To validate the outcome of the proposed workflow, we performed conventional imputation of the quality controlled and aligned genotype data of each cohort as described for the two-step imputation, with the same parameters adjusted for imputation.

### 2.5 Conventional imputation with overlapping genotypes

To evaluate a simple approach for removing the array type specific batch effect, we ran a single-step imputation by restricting the quality controlled input variants to those assessed on all array types. This approach was tested for three (Affymetrix SNP 6.0, Illumina Omni 2.5 and Illumina GSA) and all five array types, respectively. We compared the imputation quality of these imputations to the conventional and the two-step imputation exemplarily for the SHIP-TREND cohort.

### 2.6 Batch effect assessment

Technical bias was assessed by estimating genetic principal components of the imputed and quality-controlled genotype data. Quality control filters included missing variant call rates above 5%, Hardy-Weinberg equilibrium *P* values less than 10^−4^, minor allele frequency (MAF) less than 1%, correlated variants were removed by LD pruning with a window size of 50, step size of 5 SNVs and *R*^2^ threshold of 0.2. Genetic principal components, along with their explained variances were compared between two-step imputation and conventional imputation. To check the robustness of the developed imputation approach against the rare variants, we further ran PCA on rare variants (MAF < 1%). All PCAs are performed using PLINK 1.9 software (Chang *et al.* 2015). Principal components (PC) of the first 20 components were plotted with ggplot2 package of R software.

### 2.7 Evaluation of imputation performance

Genotype data attributes, including differences in MAF and imputation quality *R*^2^ measure between the two-step and conventional imputation were compared for each included cohort after stratification by minor allele frequency. We additionally checked the genetic concordance, represented by the percentage of the number of matching genotypes/total number of genotypes (Wuttke *et al.* 2023) of the imputed best-guess (hard call) genotypes with their whole-genome sequencing data of a subset of 192 individuals from SHIP-TREND (Omni 2.5) cohort for which whole genome sequencing (WGS) data was also available. To check the possibility of technical error leading to variation in certain allele combinations, the concordance results are categorized by the nature of variant’s representation into homozygous reference (HomRef), heterozygous (Het), and homozygous alternate (HomAlt).

### 2.8 GWAS and results comparison

We evaluated the results of GWAS analyses using genotype dosage information from both two-step and conventional imputation approaches using thyroid gland volume and goiter risk as outcomes as described before (Teumer *et al.* 2011), and summarized in the Methods, available as supplementary data at *Bioinformatics Advances* online.

All GWAS were conducted with the EPACTS 3.3.2 software (Kang 2016). Inverse-variance weighted meta-analysis of the single cohort GWAS summary statistics based on the conventional imputation approach was done using the METAL software (Willer *et al.* 2010). We compared the summary statistics *P* values, effect estimates and its standard errors. A detailed description of the GWAS analysis plan is available in the Methods, available as supplementary data at *Bioinformatics Advances* online. Independent (lead) variants associated with thyroid traits were identified using the clumping function of PLINK with a threshold of *P* < 5 × 10^−8^, *r*^2^ > 0.01 and 1 Mb distance, and compared across the imputation approaches. For biological validation of the findings, lead variants were investigated by checking their association with thyrotropin to support their biological plausibility (Sterenborg *et al.* 2024).

## 3. Results

Genotype datasets of 10 647 individuals genotyped on five different array types were included in the workflow (Fig. 1). After the intermediate imputation, 1 942 499 high-quality overlapping autosomal variants were generated for each of the included five datasets, and subsequently used for the second imputation step against the 1000G reference panel. In contrast, only 58 091 autosomal genotyped variants were available as imputation input in the three cohorts using the single imputation approach with overlapping variants. This number further dropped to 12 265 variants when including the Affymetrix Axiom and Illumina PsychArrays. Table 1, available as supplementary data at *Bioinformatics Advances* online, provides the distribution of these variants for each chromosome in the two-step imputation, the conventional imputation and single-step using overlapped variants. Following the imputation against 1000G panel, we produced 47 154 431 autosomal variants for the combined included cohorts using the two-step imputation approach, and 47 155 992 variants for the single imputation approach.

**Table 1.** Cohort characteristics for GWAS analysis of thyroid volume and goiter risk.^a^

Cohort	*N*	Age (mean ± SD)	Sex	BSA (mean ± SD)	Current smoker	Log thyroid volume (mean ± SD)	Goiter cases (%)
SHIP START	3611	49.1 (16.3)	47.4% male, 52.6% female	1.9 (0.2)	31.6%	2.9 (0.5)	1322 (36.6%)
SHIP-TREND	784	49.2 (13.9)	51.5% male, 48.5% female	1.9 (0.2)	22.2%	2.9 (0.4)	253 (32.3%)
SHIP-TREND batch II	2499	51.3 (16)	56.9% male, 43.1% female	1.9 (0.2)	29.8%	2.9 (0.4)	737 (29.5%)

a BSA, body surface area.

### 3.1 Batch effect investigation (PCA analysis)

**Figure 2.**
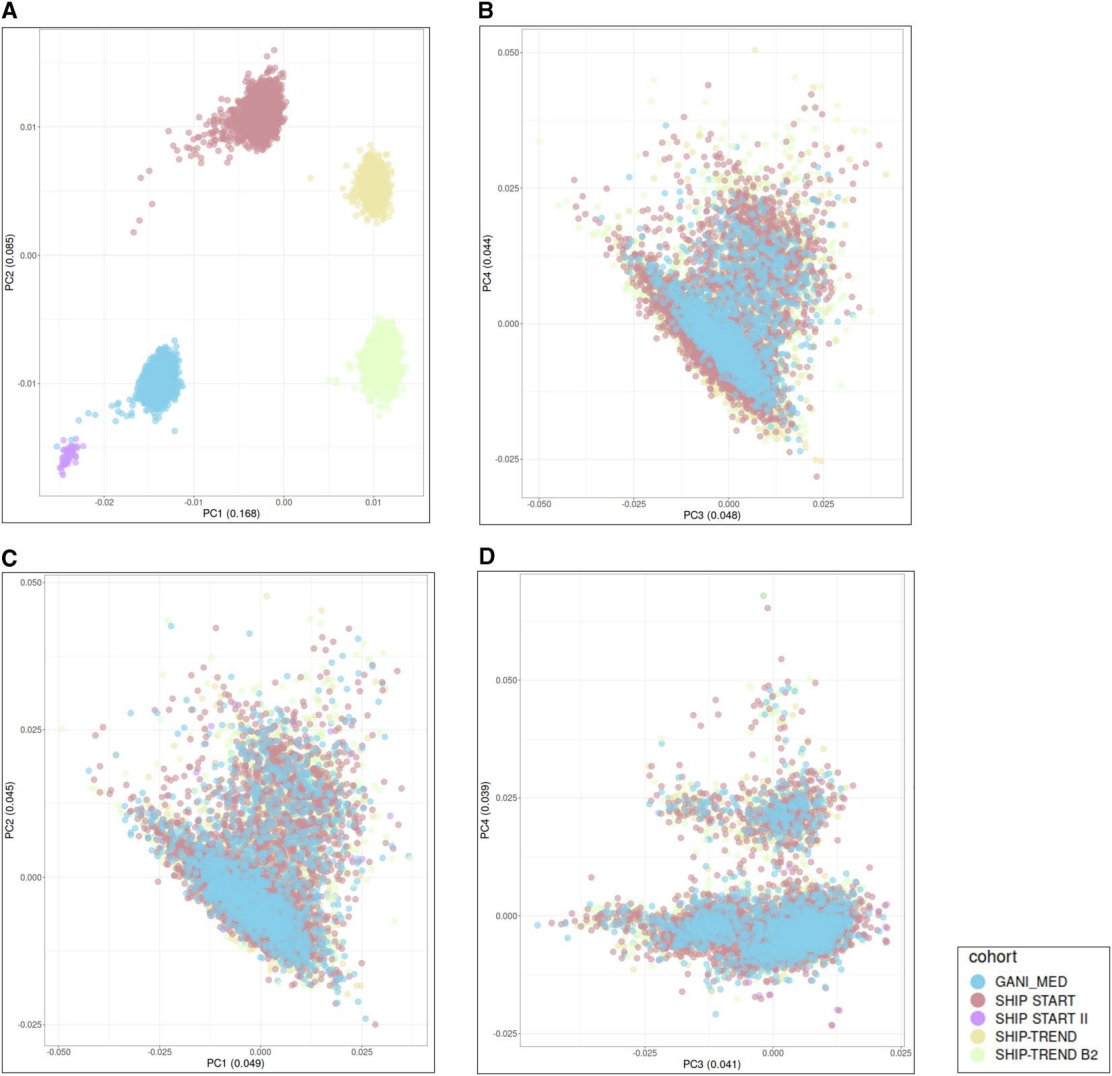
Genetic PCA of the included cohorts in the workflow. The samples are colored by the cohorts with their unique array type. Panel A and B show the first four genetic components of the conventional imputation approach (variance explained for the components combined is 0.34). Panels C and D show the first four components of the proposed approach (variance explained for the components combined is 0.17).

For the conventional imputation, 939 802 variants with a total genotyping rate of 0.988 were included in the PCA analysis, with 2 314 205 variants removed due to missing genotype threshold, 9118 variants due to Hardy-Weinberg equilibrium, and 38 008 458 variants due to the MAF threshold. Projecting the first two principle components of the imputed genotypes clustered the samples by array types with a combined explained variance of 0.242 (Fig. 2a and b, Fig. 1a, available as supplementary data at *Bioinformatics Advances* online).

The two-step imputation included 1 360 834 variants with a total genotyping rate of 0.989 in the PCA analysis after removing 725 008 variants due to missing genotype threshold, 5363 variants due to Hardy-Weinberg equilibrium, and 37 753 448 variants due to the MAF threshold. Using this approach, the first 20 principal components of the imputed genotypes did not show any array type specific clusters (Fig. 2c and d, Fig. 1b, available as supplementary data at *Bioinformatics Advances* online). PCA restricted to the rare genotypes imputed with the two-step approach did not show clustering by the array type (Fig. 2, available as supplementary data at *Bioinformatics Advances* online).

**Figure 3.**
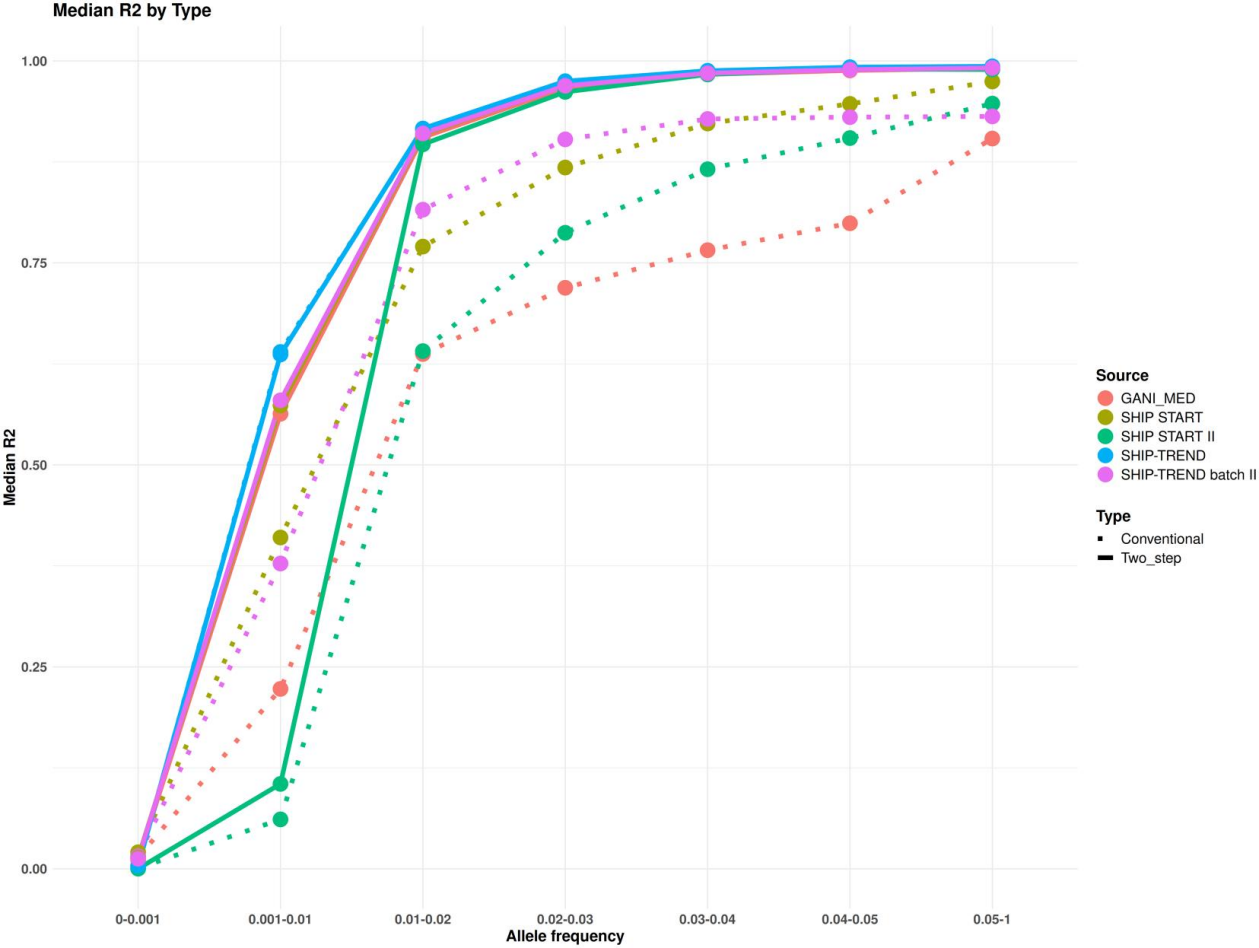
Median *R*^2^ of the imputation outcomes of genotype data of the included cohorts, using conventional (dotted) and two-step imputation (full line).

**Figure 4.**
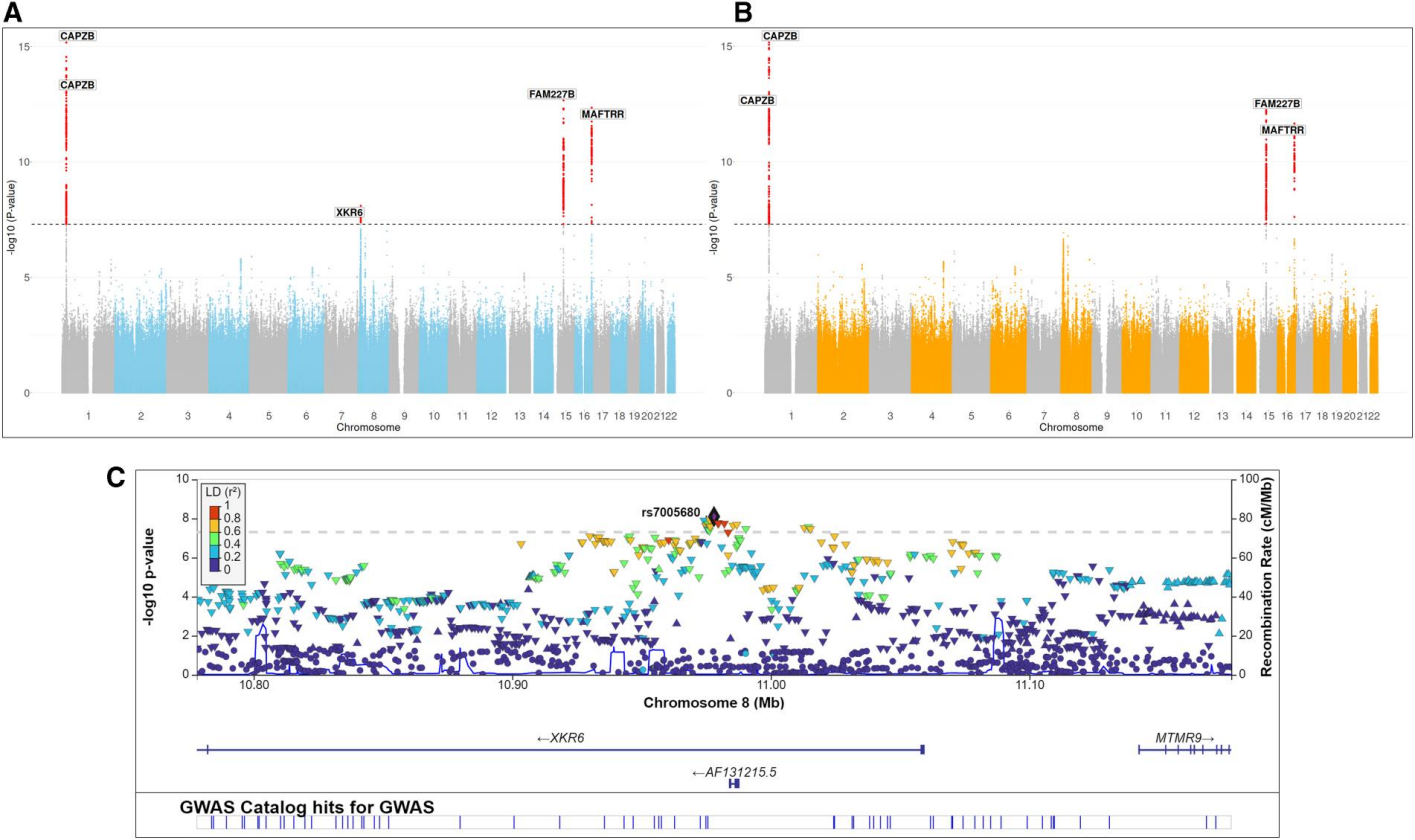
Manhattan plot of the GWAS analysis of goiter risk using combined two-step imputation genotypes (A) and conventional imputation and meta-analysis approach (B). Variants are plotted on the *x* axis and –log_10_  *P* values of the association testing on the *y* axis. Associations significant after correction for multiple testing (*P* < 5 × 10^−8^) are colored in red. Regional association results and recombination rates for the XKR6 locus are presented in part C, −log_10_  *P* values (*y*-axis) of the single nucleotide variants according to their chromosomal positions (*x*-axis) with lead variant rs7005680 is shown as a diamond.

The conventional imputation using only overlapping variants also removed the batch effect (Fig. 3, available as supplementary data at *Bioinformatics Advances* online), but led to a drastic decrease in the quality of the imputed variants for all allele frequency groups in comparison to the other approaches (Fig. 4, available as supplementary data at *Bioinformatics Advances* online). Median *R*^2^ of the overlapping variants was 0.011, while for two-step imputation and conventional imputation using all variants was 0.993 for variants with MAF > 0.05, which was the main reason for not considering this approach any further in the performance evaluation and GWAS analysis, focusing only on the two-step imputation against conventional imputation using all variants.

### 3.2 Two-step imputation outcome parameters comparison with conventional imputation

**Figure 5.**
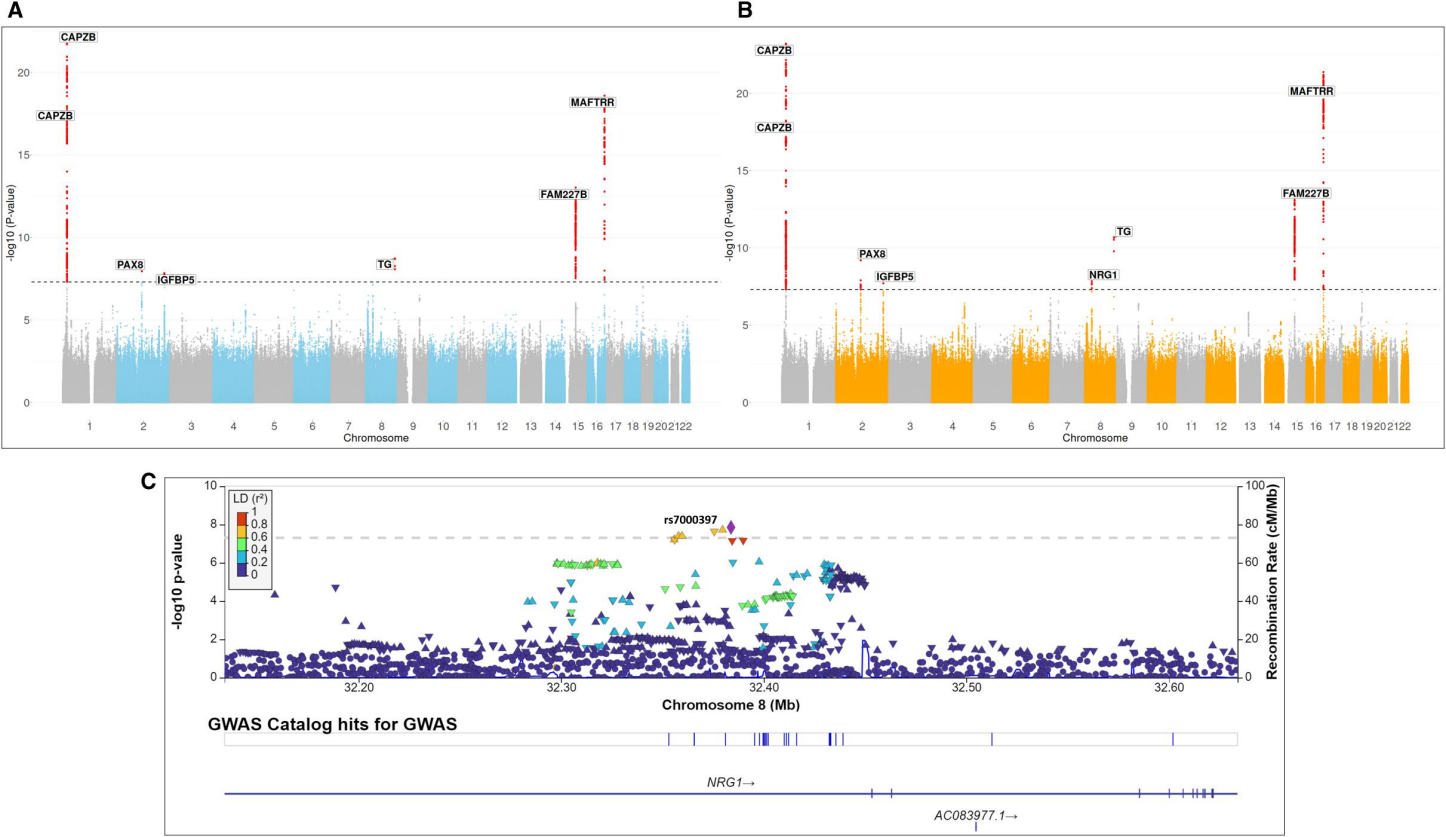
Manhattan plot of the GWAS analysis of log thyroid volume using combined two-step imputation genotypes (A) and conventional imputation and meta-analysis approach (B). Variants are plotted on the *x* axis and –log_10_  *P* values of the association testing on the *y* axis. Associations significant after correction for multiple testing (*P* < 5 × 10^−8^) are colored in red. Regional association results and recombination rates for the NRG1 locus are presented in part C, −log_10_  *P* values (*y*-axis) of the single nucleotide variants according to their chromosomal positions (*x*-axis) with lead variant rs7000397 is shown as a diamond.

Our newly developed two-step imputation approach showed higher overall quality of imputation outcome for rarer variants than the conventional imputation (Fig. 3, Fig. 5, available as supplementary data at *Bioinformatics Advances* online). For less-common variants with MAF between 0.04 and 0.05, the median *R*^2^ increased by 0.19 when using the two-step imputation approach for the GANI_MED Illumina PsychArray. It also shows comparable allele frequencies to the conventional approach, judging by the absolute difference in allele frequencies for each imputed variant in both approaches (Fig. 6, available as supplementary data at *Bioinformatics Advances* online). Detailed statistics about median *R*^2^ and allele frequency differences are presented in Table 2, available as supplementary data at *Bioinformatics Advances* online.

**Table 2.** Loci lead SNVs with the strongest association with thyroid volume, and goiter risk in GWAS analysis using both two-step imputed and conventionally imputed genotypes.^a^

Thyroid volume		Two-step imputation GWAS							Conventional imputation GWAS meta-analysis						
Locus	Lead SNP	CHR	A1	AF	Effect	SE	*P* value	Lead SNP	CHR	A1	AF	Effect	SE	*P* value	*R* ^2^
CAPZB	rs4911994	1	A	0.64	0.057	0.01	1.63E−18	rs4911994	1	A	0.63	0.055	0.01	5.93E−19	Same SNP
	rs10799824	1	A	0.15	0.086	0.01	1.82E−22	rs12410532	1	T	0.14	0.092	0.01	6.14E−24	0.978
**PAX8**	rs7560701	2	C	0.48	−0.036	0.01	1.12E−08	rs1110839	2	T	0.48	0.038	0.01	6.35E−10	0.576
**IGFBP5**	rs2712172	2	A	0.27	0.041	0.01	1.46E−08	rs2712172	2	A	0.26	0.039	0.01	1.95E−08	Same SNP
**NRG1**	rs7000397	8	G	0.35	0.036	0.01	5.22E−08	rs7000397	8	G	0.34	0.036	0.01	1.42E−08	Same SNP
**TG**	rs114322847	8	T	0.03	0.122	0.02	1.90E−09	rs79676842	8	T	0.03	0.135	0.02	2.06E−11	1
FAM227B	rs17477923	15	C	0.26	0.054	0.01	9.55E−14	rs73398264	15	T	0.75	−0.052	0.01	7.73E−14	1
MAFTRR	rs3813579	16	A	0.53	0.056	0.01	2.57E−19	rs3813579	16	A	0.52	0.056	0.01	2.67E−21	Same SNP

Goiter		Two-step imputation GWAS							Conventional imputation GWAS meta-analysis						
Locus	Lead SNP	CHR	A1	AF	Effect	SE	*P* value	Lead SNP	CHR	A1	AF	Effect	SE	*P* value	*R* ^2^
CAPZB	rs4911994	1	A	0.63	0.294	0.04	8.89E−14	rs4911994	1	A	0.63	0.302	0.04	1.12E−13	Same SNP
	rs10799824	1	A	0.15	0.411	0.05	6.68E−16	rs12410532	1	T	0.14	0.450	0.06	1.23E−15	0.978
**XKR6**	rs7005680	8	T	0.54	−0.218	0.04	7.98E−09	rs34208825	8	T	0.48	0.206	0.04	1.16E−07	0.760
FAM227B	rs75929244	15	T	0.24	0.321	0.04	2.17E−13	rs11639423	15	A	0.23	0.326	0.05	5.73E−13	0.897
MAFTRR	rs562609617	16	GT	0.35	0.290	0.04	4.44E−13	rs3813582	16	T	0.67	−0.283	0.04	2.21E−12	0.945

a Novel loci are marked in bold. A1, coded allele, AF, frequency of coded allele, *R*^2^, linkage disequilibrium in the top SNVs of the same locus in both imputation outcomes; SE, standard errors.

Hard call genotype concordance results in the subgroup of SHIP-TREND cohort with matching WGS data showed strong correlation between two-step imputation and sequenced genotypes. The Pearson correlation coefficients with the homozygous reference, heterozygous, and homozygous alternative genotypes were 0.996, 0.981, and 0.979, respectively. There were no signs that technical error affected the concordance results when we stratified the variants by their frequency or their imputation quality (Tables 3 and 4, available as supplementary data at *Bioinformatics Advances* online).

### 3.3 GWAS on thyroid traits

A total number of 6894 individuals from SHIP-START and SHIP-TREND (both array types) with thyroid measurement information were included in the GWAS analysis, all participants were of European ancestry. Detailed information about cohort characteristics is presented in Table 1. The estimated genomic control for all conducted GWAS analysis showed no signs of inflation, with a minimum and maximum ʎ_GC_ = 1.001 and 1.038, respectively (Fig. 7, available as supplementary data at *Bioinformatics Advances* online).

Meta-analysis of the GWAS analysis on goiter risk using conventionally imputed genotypes revealed four significantly associated loci (*P* < 5 × 10^−8^). Confirming the results from previously conducted GWAS analysis (Teumer *et al.* 2011). Two of the loci are located at the *CAPZB* region in chromosome 1, the other two at the *FAM227B* and *MAFTRR* regions on chromosome 15 and 16, respectively (Fig. 4b). However, the GWAS analysis of the combined two-step imputed genotype data revealed another associated locus at the *XKR6* region on chromosome 8 (Fig. 4c), which did not reach statistical significance using the conventional meta-analysis approach (Fig. 4a). The associated variant at *XKR6* was not genotyped on any array, and thus directly imputed against 1000G reference panel in both imputation approaches.

The conventional approach of the GWAS meta-analysis for thyroid volume revealed four novel associations at the *PAX8* region on chromosome 2, at *IGFBP5*, *NRG1* and *TG* (Fig. 5b and c), and confirmed the known associations with this trait (Sterenborg *et al.* 2024). Genome-wide significance for these regions were also obtained with the GWAS using the combined two-step imputed genotype data, with the exception of *NRG1* (*P* value = 5.22 × 10^−8^) (Fig. 5a). This variant was directly genotyped on all array types except the Illumina GSA used for SHIP-TREND batch II, meaning that it was intermediately imputed for that sample. Except the *PAX8* region, all associated loci were also associated with thyroxin in recently published multi-trait GWAS meta-analysis analysis for thyroid function (Sterenborg *et al.* 2024).

Table 2 summarizes the results of the SNVs with the strongest association of both GWAS approaches and traits. The significant GWAS results of both approaches were comparable, judging by the magnitude of the estimates. However, the standard errors of the GWAS results obtained from the two-step imputed genotypes where generally lower, where the p-values were slightly higher in the linear regression and lower in the logistic regression based analyses compared to the conventional imputation and subsequent meta-analysis (Figs. 8 and 9, available as supplementary data at *Bioinformatics Advances* online).

## 4. Discussion

Genome-wide association studies have helped identify genetic factors for many traits and diseases. Since the 1960s, collecting genotype samples from diverse populations has grown in importance (Evaluating Human Genetic Diversity 1997, Nelson *et al.* 2008). Imputation techniques now address gaps in whole-genome sequencing (Treccani *et al.* 2023). However, variations in these methods across cohorts reduce the accuracy of genetic analyses due to biases from differing array technologies (Lachance and Tishkoff 2013, Geibel *et al.* 2021). For instance, in our genotyped samples where we compared the allele frequencies of the SHIP-TREND subgroup genotyped using Illumina Omnia 2.5 with their whole genome sequencing (Fig. 10, available as supplementary data at *Bioinformatics Advances* online), this array type specific variation in the allele frequency, both in common and rare variants, can affect LD estimation in haplotype phasing and genotype imputation. However, our developed imputation method addresses the additional variation induced by including multiple array types. By forming an intermediate panel of high-quality variants, our method enables high imputation accuracy while removing the array type induced batch effects.

Although the complexity of the first imputation step scales quadratically with the number of different array types, there are several aspects to consider regarding the overall runtime. First, the number of imputed variants in this step is limited by the number of variants of the respective array type which is substantially lower than the common reference panel like 1000G or HRC included in step 2. In detail, the phasing and imputation of our dataset encompassing 10 647 individuals and five different array types took 27 hours parallelizing on only twelve 2.8 GHz Xeon CPU cores. Second, additional samples that were genotyped using one of the already existing array types need to be imputed only to the intermediate imputation panel already generated in step 1 before their final imputation to the common reference panel in step 2. Finally, only step 2 has to be re-run when changing the common reference panel, e.g. from 1000G to TOPMed.

The developed workflow was inspired by the use of only overlapping variants in the included cohorts (Table 1, available as supplementary data at *Bioinformatics Advances* online), which eliminated the observed bias, yet led to a significant decrease in the imputation quality due to removal of informative tag SNVs (Fig. 3, available as supplementary data at *Bioinformatics Advances* online), the influence of informative SNVs on imputation quality has been shown in previous research work (Wojcik *et al.* 2018). Based on that, we introduced an intermediate imputation step to generate a panel that can retain the same genotype information of the included arrays and thus preserving its LD structure. We strongly assume that the observed batch effect is caused by the underlying SNV selection and density included in the respective array design which covers different LD structures of the genotyped individuals. Thus, we included an intermediate imputation step based on genotyping information from the same underlying population to improve the accuracy and consistency of the final imputation results. This step enables more precise haplotype reconstruction by first imputing within a population-specific reference panel, thereby reducing potential imputation errors when integrating data from different arrays. Additionally, it minimizes batch and platform effects likely arising from variations in SNV representation across different genotyping arrays, harmonizing these discrepancies and generating a uniform set of high-confidence variants for the subsequent imputation step.

Selecting an appropriate threshold for the imputation *R*^2^ of the overlapping variants in the intermediate panel was essential for having reliable genotype information upon imputing against 1000G panel. The value of the threshold was decided following the PCA output of several imputations using different thresholds. We aimed to use the highest possible value for imputation quality *R*^2^ without affecting haplotype phasing results due to removing too many variants. Table 1, available as supplementary data at *Bioinformatics Advances* online, shows the number of included variants per chromosome when the *R*^2^ threshold was adjusted to 0.8 as well as 0.9. Both approaches led to similar imputation outputs as seen after plotting genetic PCs of the imputation outcomes (Fig. 2 and Fig. 11, available as supplementary data at *Bioinformatics Advances* online).

To evaluate the existence of the array type specific imputation bias, we used genetic PCs to capture the main variance in the allele frequencies of the imputed genotypes, which can be seen as the influence of the array type after projecting the eigenvectors and evaluate the homogeneity of the projected points on PC axis (Elhaik 2022). The imputation following the developed two-step approach showed its capability to overcome the array type differentiation whenever existing in the first twenty components, compared to conventional imputation outcomes where a clear clustering effect by the array type was observed (Fig. 2).

To test the performance of the imputation outcomes, we focused on comparing allele frequency and imputation quality parameters of the cohort genotypes. These parameters are particularly relevant for conducting trait-association studies. The developed imputation flow showed strong matching of the allele frequency, represented by minimized difference in allele frequency for each variants between the imputation approaches (Fig. 6, available as supplementary data at *Bioinformatics Advances* online). The developed imputation workflow was successful in providing more reliable genetic predictions for imputed rare alleles (Fig. 3). In contrast to the other cohorts, a strong concordance of the median *R*^2^ between both imputation approaches was observed in SHIP-TREND. This could be due to the high variant coverage of the array used for genotyping the cohort’s samples. Further analysis of the allele frequency variance for each variant from both imputation approaches of this specific cohort shows the strong correlation in the autosomal allele frequency (correlation *r*^2^ >0.999) (Fig. 12, available as supplementary data at *Bioinformatics Advances* online). Nevertheless, SHIP-TREND imputed genotypes showed strong concordance with the corresponding genotyped variants in the whole-genome sequenced subgroup, indicating the representation of well-estimated genotypes in both rarer and common variants. Using the allele frequencies of the WGS subgroup as a gold standard for comparison is somewhat misleading due to differences in the frequencies observed already between genotyped variants on the genotyping arrays and the WGS (Fig. 10, available as supplementary data at *Bioinformatics Advances* online).

The aim of conducting GWAS as part of the imputation workflow evaluation is to compare linear regression outcomes of the combined two-step imputed datasets to the conventional inverse-variance meta-analysis for the same samples. Goiter risk and thyroid volume are both suitable traits for our evaluation as they represent different trait datatypes with a true positive genetic association in the SHIP cohorts (Teumer *et al.* 2011). The results of GWAS analysis for the two-step imputed data showed two key advantages. Firstly, we were able to identify an association in the *XKR6* gene region with goiter risk as a dichotomous trait which did not reach statistical significance in the conventional meta-analysis. Meta-analyzing GWAS results of the two-step imputation using METAL did not show differences with the significant hits, indicating that these results are not influenced by differences in the analysis method and only due to difference in the imputation approach (Fig. 13, available as supplementary data at *Bioinformatics Advances* online). A slightly better performance of the mega-analysis vs. a meta-analysis for a logistic regression GWAS is in line with the results of a former study (Gorski *et al.* 2019). The second point was its consistency with conventional imputation GWAS meta-analysis in revealing a novel locus associated with thyroid gland volume (*PAX8*). *PAX8* is a paired box family gene member that was found to be associated with the development of thyroid gland in embryonic development, and its transcription is a diagnostic marker for anaplastic thyroid carcinoma (Bishop *et al.* 2011, Ozcan *et al.* 2011, Suzuki *et al.* 2015), and thus represents also a plausible association with thyroid volume in adults. All other significant GWAS findings represent also plausible true positive associations as they confirmed former findings of these traits (including replication in an independent cohort) (Teumer *et al.* 2011), or were associated with thyroid function (Sterenborg *et al.* 2024).The comparison of the estimates and standard errors of both GWAS approaches did not show signs of *P* value inflation. However, it showed that the GWAS of the two-step imputation data had a slight decrease in standard errors, in comparison to the meta-analysis outcomes. In general, the differences in the association results between both imputation approaches for the continuous trait GWAS (thyroid volume) are somewhat smaller compared to dichotomous trait GWAS (goiter) as indicated in Table 2 and reflected by the ʎ_GC_ (Fig. 7, available as supplementary data at *Bioinformatics Advances* online). This minor difference was also observed for the variant rs7000397 that did not reach genome-wide significance in the mega-analysis of thyroid volume (5.22 × 10^−8^ vs. 1.42 × 10^−8^). Reviewing the imputation background of rs7000397 located in *NRG1*, we found that it was directly genotyped in all but the Illumina GSA array used for the SHIP-TREND batch II cohort. This variant was intermediately imputed in the two-step imputation, while it was directly imputed against the external reference panel in this cohort in the single imputation. This likely led to the variation in the GWAS results exemplarily observed at this locus (the same lead SNV in both imputation approaches). These minor differences in the continuous trait regression results might be related to statistical variation or a slight decrease in power attributed to the additional imputation step. However, the advantage of combining the samples in a mega-analysis while removing imputation batch effects seems to outweigh such limitations when applying the two-step approach in a logistic regression as indicated by the goiter GWAS. Further investigations across additional traits and datasets are needed to better understand under which conditions each approach performs optimally. However, the main goal of our proposed approach is to minimize batch effects introduced by combining data from different sources, while preserving statistically significant GWAS signals and ensuring robustness of analysis results.

To illustrate the influence of the batch effect on a genome-wide scale, we conducted an additional GWAS analysis of our two SHIP-TREND datasets using the batch as the targeted outcome. We found that GWAS results for conventional imputation was highly inflated using all variants as well as variants with allele frequency above 0.01 (λ  =  2.61, 1.84, respectively). When using the two-step imputation as target, the inflation is removed for common and substantially reduced for low frequency variants (λ = 1.14 using all variants and 1.04 with allele frequency above 0.01). These results were virtually unchanged using best-guess genotypes in the GWAS instead of the allele dosages.

Besides its role in discovering more hits in case-control GWAS analysis, our developed two-step imputation workflow is not restricted to a specific array type. The inclusion of different arrays with different SNP density in the workflow has proven its robustness in overcoming the array type specific bias. By re-using the output of the first imputation step, the workflow is also efficiently applicable for other generalized imputation panels like TOPMed or HRC reference panels (Das *et al.* 2016). These two strengths shall enable the utilization of combined genotype information for better understanding of rarer diseases.

We applied our two-step imputation approach exemplarily on a GWAS. The array type specific batch effect in GWAS mega-analyses might be reduced in specific scenarios by adjusting for genetic PCs, or by array type stratified GWAS with subsequent meta-analysis. However, such a correction will not be possible in all analyses. Such analyses include the ones using polygenic scores (PGS) to identify individuals at risk for a specific disease. Our two-step imputation provides a powerful solution for combining datasets while reducing technical bias also for PGS analyses.

Comparing the imputation outcomes to the whole genome-sequenced data had limitations for evaluating the proposed workflow, either due to the relatively small sample size of individuals who underwent whole-sequencing (*n = *192 after QC) and its exclusivity for one of the included five cohorts. Although the total sample size provided us the possibility of analyzing low frequency alleles, it was likely too small for evaluating the performance of the different approaches for very rare alleles. However, our two-step imputation approach seems to be superior in the imputation quality measure compared to the classical imputation (Fig. 3), while the difference in allele frequency is small particularly for rarer variants (Fig. 4 and Table 2, available as supplementary data at *Bioinformatics Advances* online). As indicated also in these results, the average difference in allele frequency seems to depend also on the density and design of the underlying genotyping array.

While the imputed genotypes generated from the proposed workflow will be utilized for identifying novel genetic associations in different SHIP and GANI_MED cohorts, it will be interesting to evaluate the impact of the developed workflow on other cohorts, especially those comprising diverse ancestries. Thus far, all the included cohorts are from European ancestry from the Northeast of Germany. Highlighting the need to test the workflow’s efficacy on genotyped cohorts from other ancestries or cohorts with multiple ancestries. Such evaluations will help determine the generalizability and robustness of the imputation method across different genetic backgrounds, ultimately enhancing its utility in global genomic research.

In conclusion, our developed two-step imputation workflow aims to overcome the array type bias, by creating an intermediate panel of high-quality overlapping imputed variants. This approach enables the conduction of mega-analysis by combining genotype information from different arrays without inducing a technical array type effect. Our workflow will increase statistical power for conducting large-scale GWAS mega-analyses and other genetic analyses like polygenic risk score calculations, playing an important role in genetic research and its application in individualized medicine.

## Supplementary Material

vbaf317_Supplementary_Data

## Data Availability

The data of the SHIP study cannot be made publically available due to the informed consent of the study participants, but it can be accessed through a data application form available at https://fvcm.med.uni-greifswald.de/ for researchers who meet the criteria for access to confidential data. The full results of the GWAS summary statistics are available on the ThyroidOmics Consortium website (http://www.thyroidomics.com). Developed scripts for the workflow are available on github (https://github.com/GenEpi-psych-UMG/Two_Step_Imputation).
