# Supplementary material for: Removing array-specific batch effects in GWAS mega-analyses by applying a two-step imputation workflow
Source: Bioinform Adv. 2025 Dec 14;6(1):vbaf317. doi: 10.1093/bioadv/vbaf317 (PMC12970591; doi:10.1093/bioadv/vbaf317)
Supplement: vbaf317_Supplementary_Data [file vbaf317_supplementary_data.pdf]

# Removing array-specific batch effects in GWAS mega-analyses by applying a two-step imputation workflow

Nasr MK *et al.*

SUPPLEMENTARY INFORMATION

**Table of Contents**


Supplementary Methods ..... 3

Summary of the included cohorts ..... 3

SHIP ..... 3

GANI_MED ..... 3

Array panels included in the imputation workflow ..... 3

Affymetrix SNP 6.0 ..... 3

Affymetrix Axiom ..... 3

Illumina Omni 2.5 ..... 3

Illumina GSA ..... 3

Illumina PsychArray ..... 4

Participant information and GWAS methodology for thyroid volume and goiter risk ..... 4

Supplementary Figures ..... 5

Supplementary Figure 1. Genetic PCs (5-20) with their explained variance for conventional imputation (panel A) and two-step imputation (panel B) ..... 5

Supplementary Figure 2. Genetic PCs (1-6) with their explained variance for two-step imputation using rare variants (MAF < 0.01) ..... 6

Supplementary Figure 3. Genetic PCs (1-6) with their explained variance for conventional imputation using overlapped genotyped variants for imputation ..... 6

Supplementary Figure 4. Median $R^2$ of the imputation outcomes of different approaches. ..... 7

Supplementary Figure 5. Boxplots grid of the $R^2$ of the imputed variants and coloured by the imputation type. ..... 8

Supplementary Figure 6. Boxplots grid of the absolute difference in AF between conventional and two-step imputation outcomes. ..... 9

Supplementary Figure 7. Quantile-Quantile plots of the GWAS results ..... 10

Supplementary Figure 8. Comparison of the goiter risk GWAS summary statistics ..... 11

Supplementary Figure 9. Comparison of the log thyroid volume GWAS summary statistics ..... 11

Supplementary Figure 10. Genotyped versus sequenced allele frequencies ..... 12

Supplementary Figure 11. Genetic PCs (1-4) with the explained variance for two-step imputation using an imputation quality threshold for intermediate imputation $R^2 > 0.8$ ..... 12

Supplementary Figure 12. Comparison of the minor allele frequency of the SHIP-TREND imputation ..... 13

Supplementary Figure 13. Manhattan plot of the GWAS analysis of goiter risk using combined two-step imputation genotypes meta-analysed by METAL. ..... 14

Supplementary Tables ..... 15

Supplementary Table 1. Distribution of the included variants for imputation against 1000G reference panel in the proposed two-step imputation with an $R^2$ threshold of 0.8 and 0.9 respectively. ..... 15

Supplementary Table 2. Differences in median $R^2$ between conventional and two-step imputation approaches ..... 16

Supplementary Table 3. Genotype concordance with sequenced data stratified by allele frequency. ..... 17

Supplementary Table 4. Genotype concordance with sequenced data stratified by imputation quality $R^2$. ..... 17

References ..... 17

## Supplementary Methods

### Summary of the included cohorts

*SHIP*

The Study of Health in Pomerania (SHIP-START and SHIP-TREND) are parts of the Community Medicine Research network of the University of Greifswald, Germany, which are funded by the multiple institutions (grants no. 01ZZ9603, 01ZZ0103, 03Z1CN22, and 01ZZ0403), SHIP is a population-based project conducted in West Pomerania, a region in the northeast of Germany, consisting of two independent, prospectively collected cohorts (SHIP START and SHIP-TREND), with an objective of assessing the prevalence and incidence of common population-based diseases and their associated risk factors (Völzke *et al.*, 2011). A sample of the region's population who have German citizenship and main residency was randomly drawn and stratified by age and gender. Baseline examinations were carried out for SHIP START from 1997 until 2001, resulting in a sample of 4,308 participants. While the baseline examinations for SHIP-TREND were carried out between 2008 and 2012, with 4,420 individuals included.

*GANI_MED*

The research project "Greifswald Approach to Individualized Medicine" (GANI_MED) is a cohort with a focus on cardiovascular, cerebrovascular and metabolic diseases. The primary aim of this cohort is to increase the number of therapeutic strategies in personalized medicine approach (Grabe *et al.*, 2014). Official patient recruitment started in 2011 with more than 4000 individuals included in the study.

### Array panels included in the imputation workflow

*Affymetrix SNP 6.0*

The Affymetrix SNP 6.0 array contains more than 906,600 single nucleotide variants (SNVs) presented on 200 to 1,100 base pairs. SNVs are amplified using the Genome-Wide Human SNP Nsp/Sty Assay Kit 5.0/6.0, which is also validated for use for whole-genome sampling assay. This array was utilized for genotyping individuals from SHIP START cohort, comprising 4,070 individuals who were genotyped and subjected to quality control (QC) filtering.

*Affymetrix Axiom*

Axiom array was used for genotyping subgroup of 48 individuals from SHIP START cohort. This array features 560,000 SNVs available on the array, mostly available in HapMap. Two rounds of SNV selection were conducted with separate set of SNVs. The sit is considered to be of important biological value.

*Illumina Omni 2.5*

With more than 2.4 million SNVs per array, the Omni 2.5 delivers the most comprehensive coverage of SNVs among the included arrays in our project. This array was used for genotyping 986 individuals from SHIP-TREND cohort.

*Illumina GSA*

The Infinium Global Screening Array (GSA) V3.0 is an advanced genotyping array which includes a multiethnic genome-wide backbone. GSA was used for genotyping 3,133 individuals from SHIP-TREND who are not overlapping with individuals genotyped with Omni 2.5 and included in our project.

*Illumina PsychArray*

The Illumina PsychArray array was designed with a focus on psychiatric disorders risks, covering more than 560,000 variants including tag SNVs from the HumanCore and Exome BeadChip, and other markers associated with common psychiatric disorders. 2,410 individuals from GANI_MED cohort who were genotyped by this array were included in the project.

**Participant information and GWAS methodology for thyroid volume and goiter risk**

We included European ancestry participants from three SHIP cohorts. A total of 6,894 individuals, aged 20 to 83, have been included from SHIP START, SHIP-TREND and SHIP-TREND batch II for association testing. GWAS was conducted twice for each investigated trait. One with combined two-step imputed genotypes. And another one using conventionally imputed genotypes in each cohort separately, followed by meta-analysing the summary statistics of the three included cohorts.

All included individuals have a valid thyroid volume measurement determined by ultrasonography using an Ultrasound VST-Gateway (Diasonics, Santa Clara, USA) beside their quality controlled genotype data. To eliminate the confounding effect, we excluded individuals who are diagnosed with thyroid disorders or taking thyroid medication. Pregnant individuals were also excluded from the analysis. Goiter risk was identified as a dichotomous variable from the thyroid volume, all individuals with a thyroid volume above 25 and 18 ml for males and females respectively were identified with goiter risk. We used the natural log transformed values of the thyroid gland's volume for GWAS analysis. Only autosomal variants with a minor allele frequency above 0.01 and minor allele count above 1 were included. The statistical testing was performed using EPACTS software pipeline, using linear Wald testing for quantitative thyroid volume trait, and logistic Wald testing for the goiter risk trait(Kang HM, 2016). Both models were adjusted for age, sex, current smoking status, and body surface area (0.007184*(weight in kilograms$^{0.425}$)*(height in centimetres$^{0.725}$)) as covariates. The threshold for genome-wide significance was $5 \times 10^{-8}$ after correction for genomic control when needed.

## Supplementary Figures

**Supplementary Figure 1. Genetic PCs (5-20) with their explained variance for conventional imputation (panel A) and two-step imputation (panel B)**

**A)**

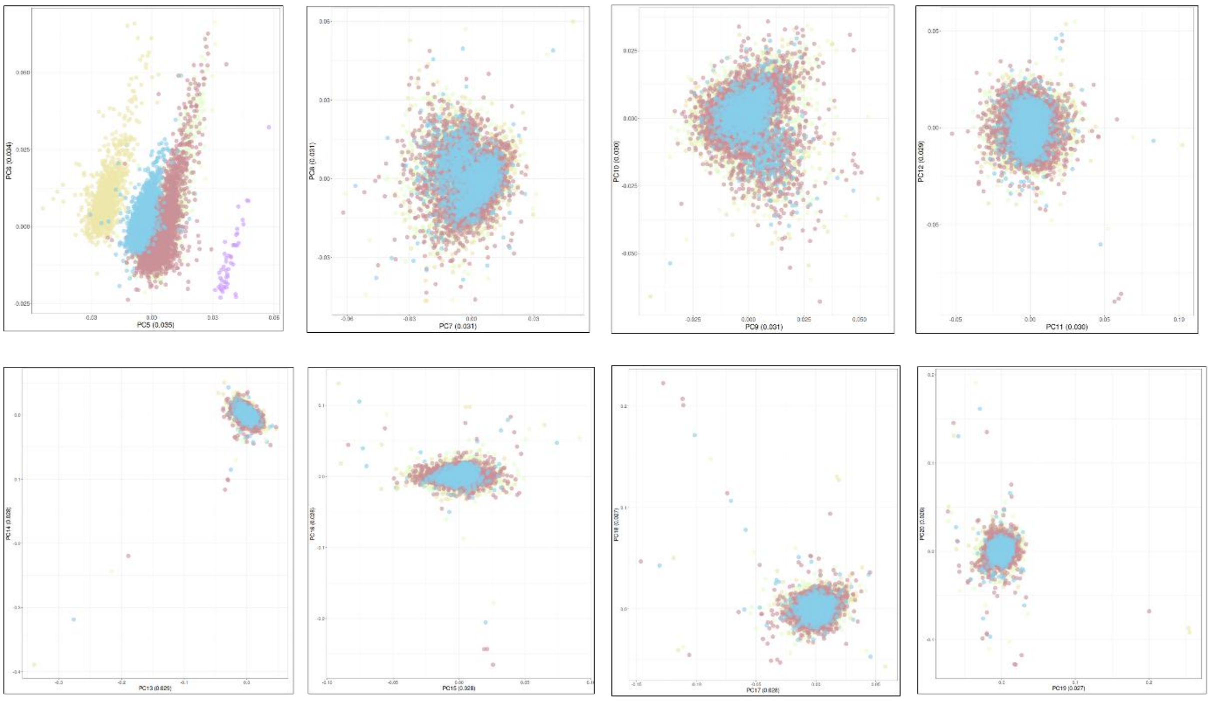

**B)**

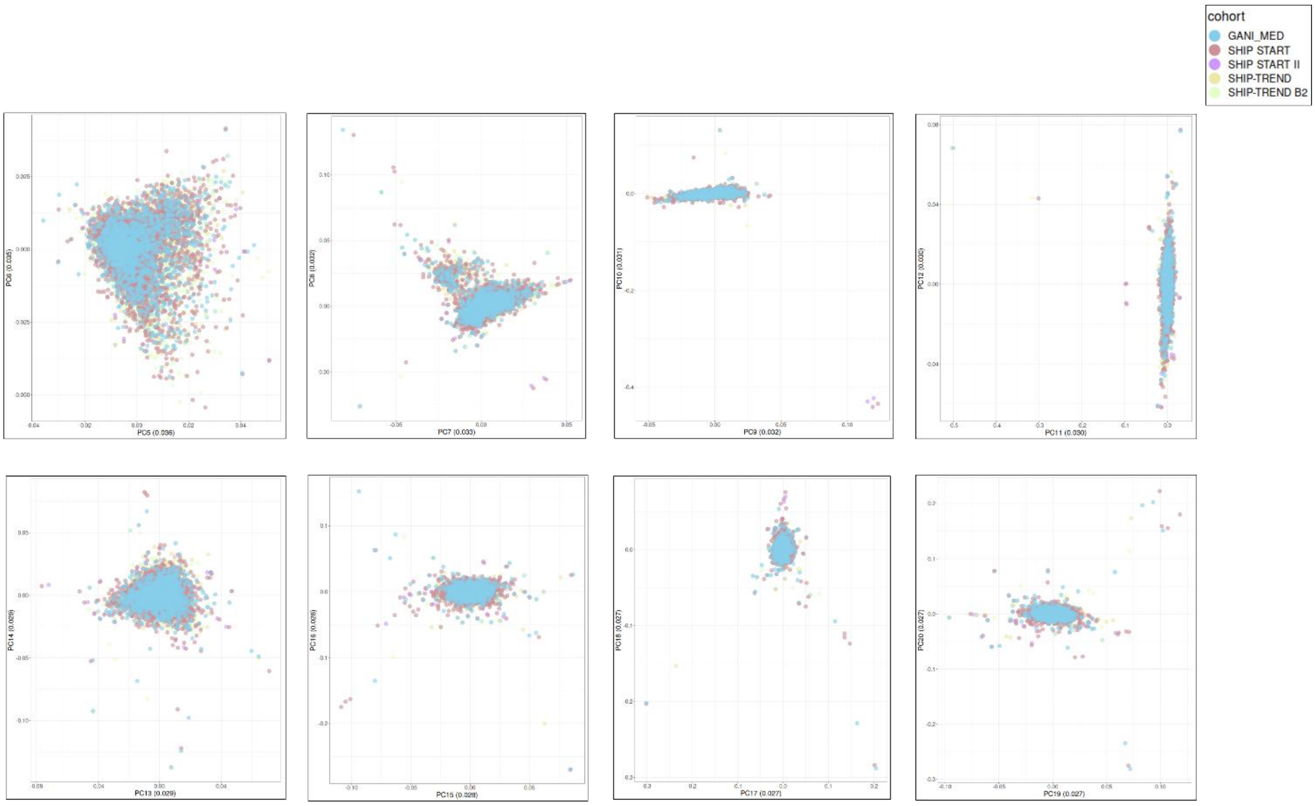


The samples are colored by the cohorts with their unique array type.

**Supplementary Figure 2. Genetic PCs (1-6) with their explained variance for two-step imputation using rare variants (MAF < 0.01)**

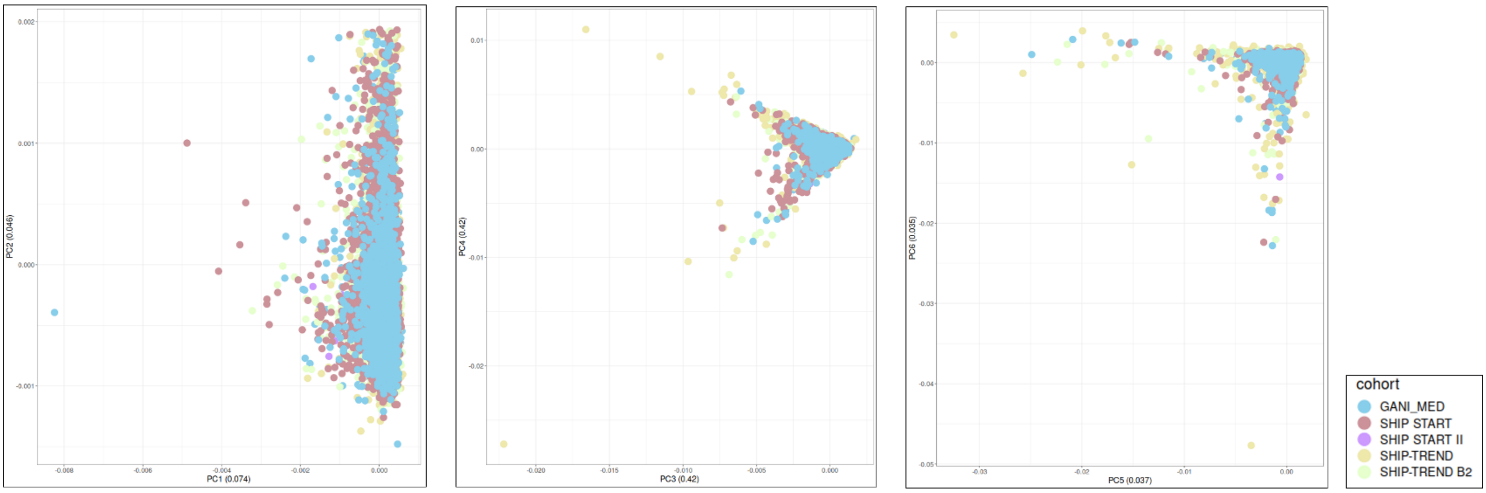


The samples are colored by the cohorts with their unique array type.

**Supplementary Figure 3. Genetic PCs (1-6) with their explained variance for conventional imputation using overlapped genotyped variants for imputation**

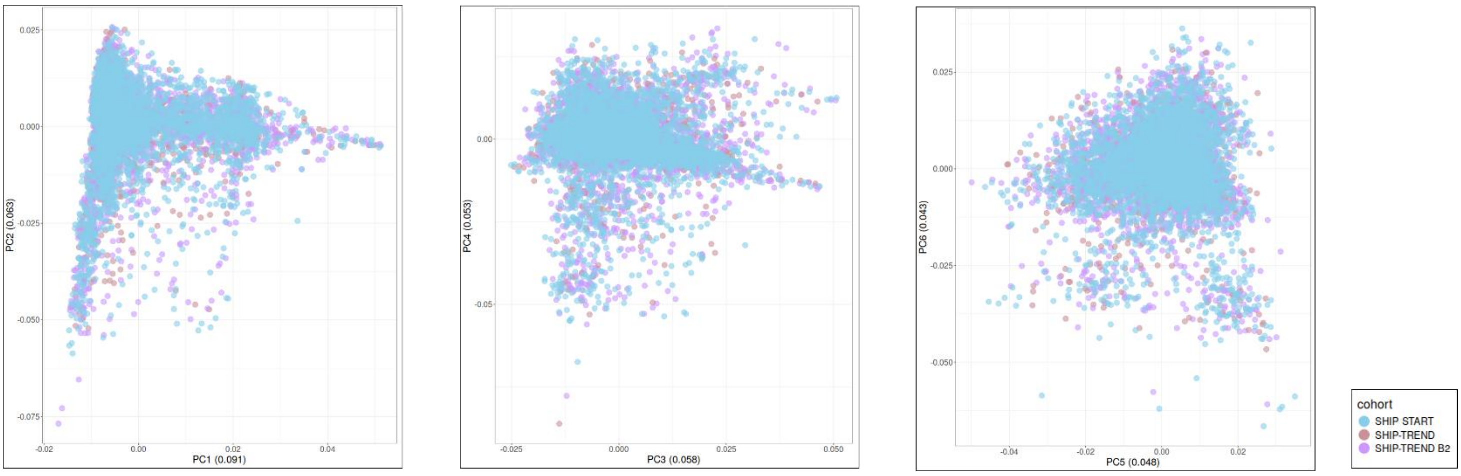


The samples are colored by the cohorts with their unique array type.

**Supplementary Figure 4. Median $R^2$ of the imputation outcomes of different approaches.**

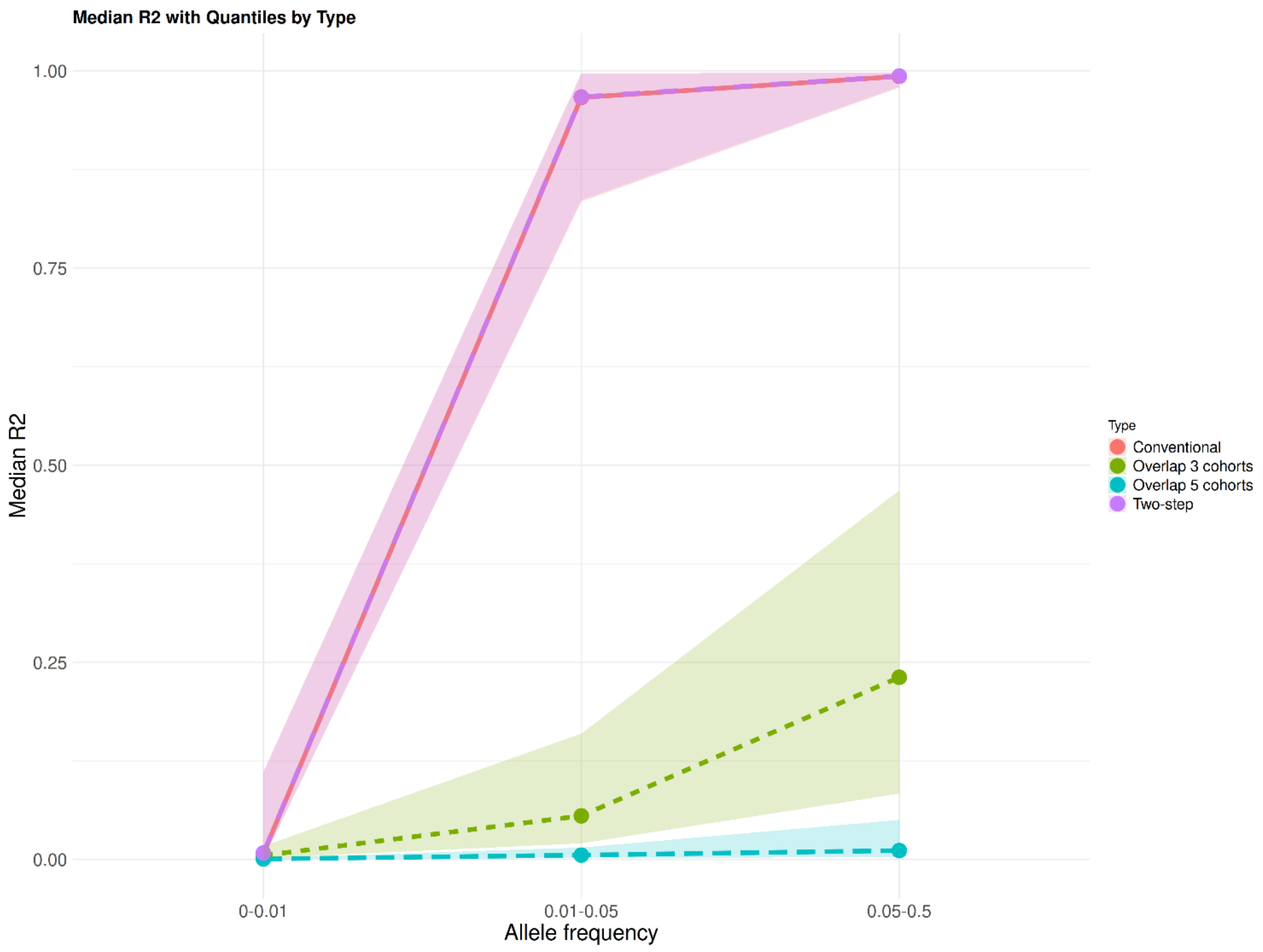


The x axis represents allele frequency group ranging from 0.001 to 0.5.

**Supplementary Figure 5. Boxplots grid of the $R^2$ of the imputed variants and coloured by the imputation type.**

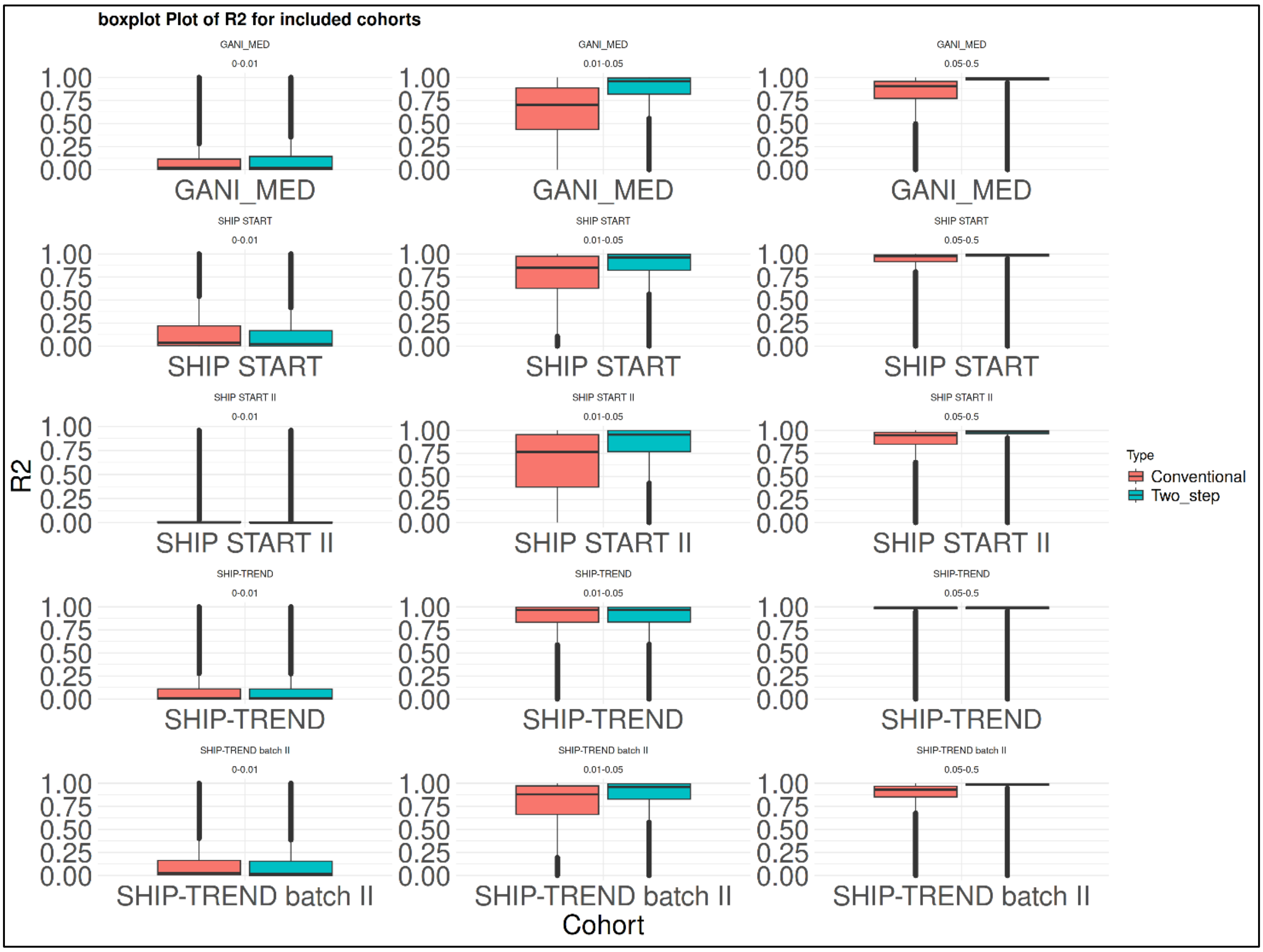


Each column represents allele frequency group (rare, low, common) and each row represents one of the included cohorts.

**Supplementary Figure 6. Boxplots grid of the absolute difference in AF between conventional and two-step imputation outcomes.**

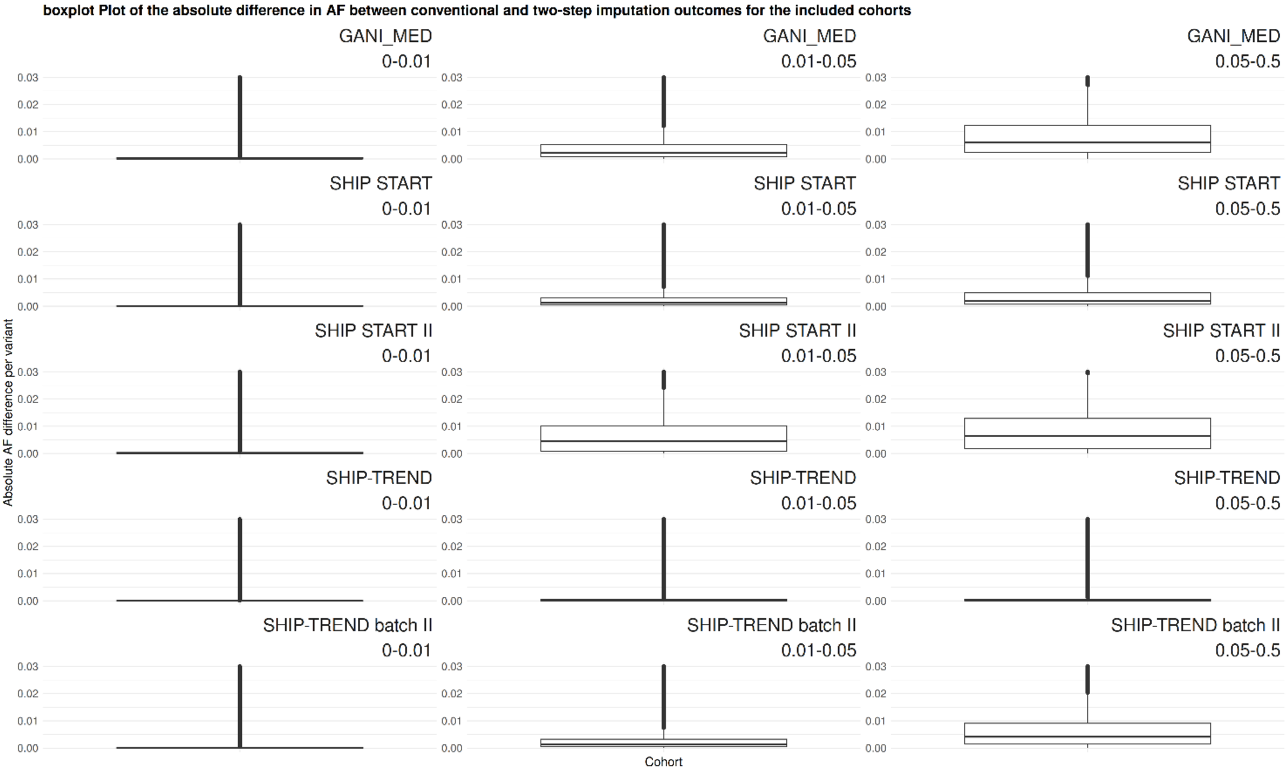


Each column represents an allele frequency group (rare, low, common) and each row represents one of the included cohorts.

**Supplementary Figure 7. Quantile-Quantile plots of the GWAS results**

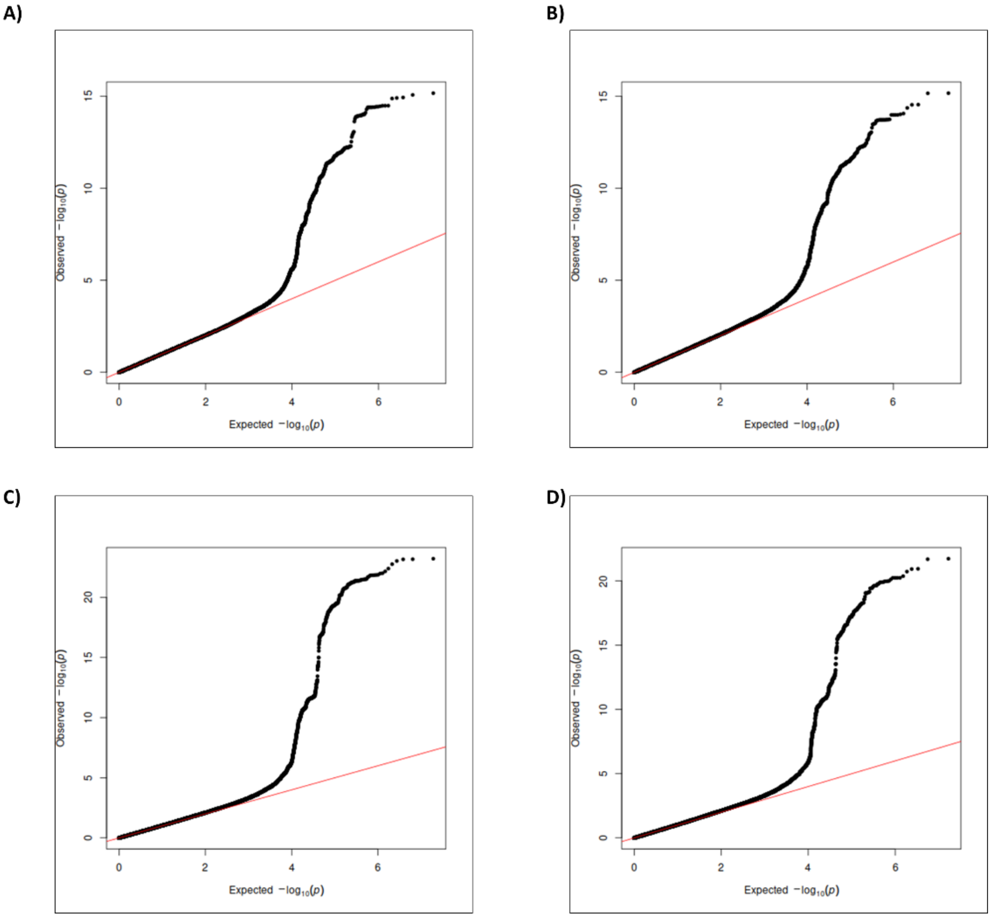


QQ plots for goiter risk using conventional imputation (panel A, $\Lambda_{GC}$ = 1.001), the combined two-step imputation (panel B, $\Lambda_{GC}$ = 1.030), and log thyroid volume using conventional imputation (panel C, $\Lambda_{GC}$ = 1.036) and the combined two-step imputation (panel D, $\Lambda_{GC}$ = 1.038)

**Supplementary Figure 8. Comparison of the goiter risk GWAS summary statistics**

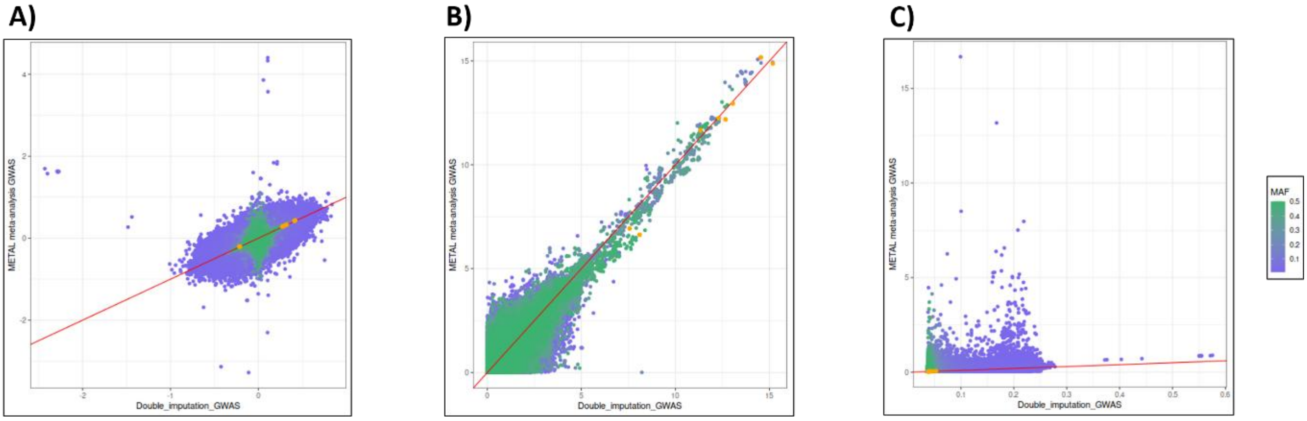


Scatter plots of effect estimates, -log$_{10}$ p-values and standard errors (panels A, B and C respectively) for the GWAS based on the two-step imputation (x axis) and the meta-analysis of the conventional imputation (y axis). Points are colored by minor allele frequency (MAF) of the two-step imputed genotypes. Points colored in yellow represent the SNVs with significant association with goiter risk in both GWAS analyses.

**Supplementary Figure 9. Comparison of the log thyroid volume GWAS summary statistics**

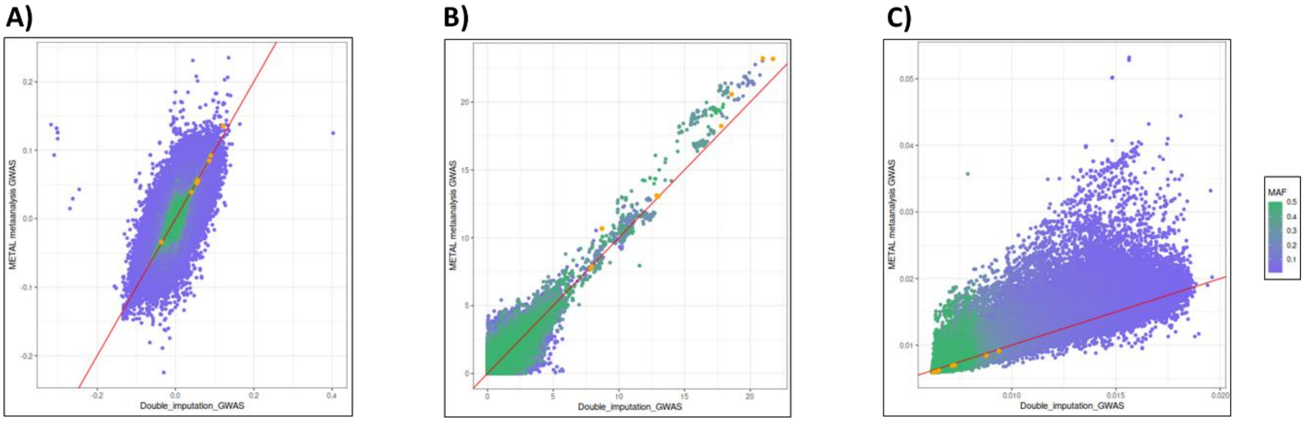


Scatter plots of effect estimates, -log$_{10}$ p-values and standard errors (panels A, B and C respectively) on the two-step imputation (x axis) and the meta-analysis of the conventional imputation (y axis). Points are colored by minor allele frequency (MAF) of the two-step imputed genotypes. Points colored in yellow represent the SNVs with significant association with log thyroid volume in both GWAS analyses

**Supplementary Figure 10. Genotyped versus sequenced allele frequencies**

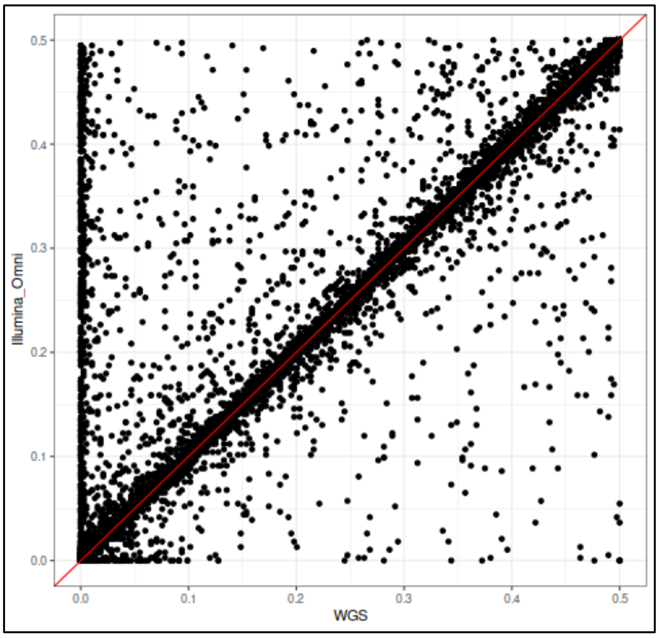


Minor allele frequencies in SHIP-TREND subgroup (n= 192) genotyped with Illumina Omni 2.5 (y axis) versus whole genome sequencing (WGS) (x axis). Variants with missing genotypes were filtered out.

**Supplementary Figure 11. Genetic PCs (1-4) with the explained variance for two-step imputation using an imputation quality threshold for intermediate imputation $R^2 > 0.8$**

**A)** **B)**

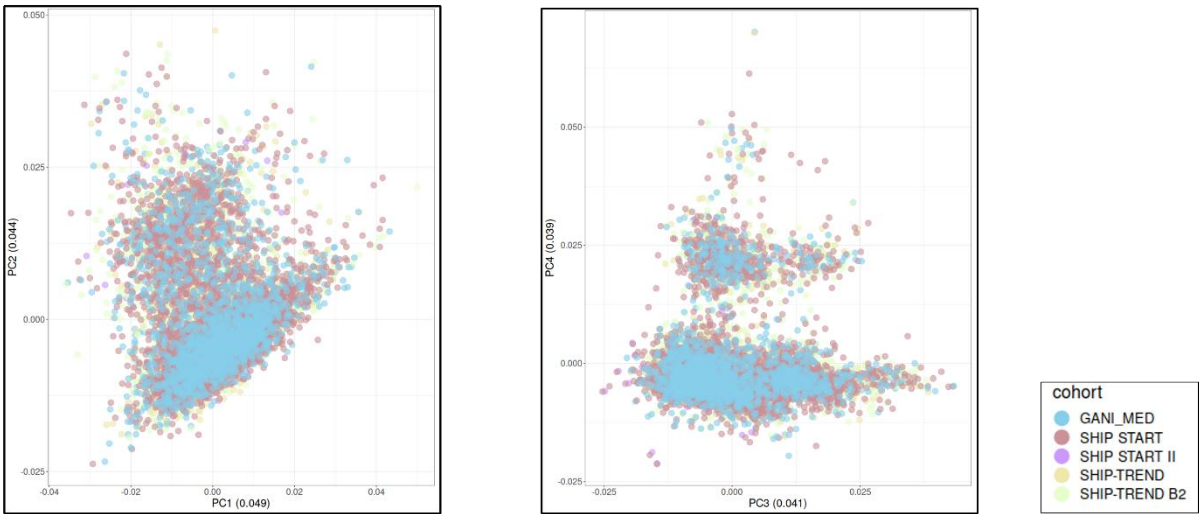

**Supplementary Figure 12. Comparison of the minor allele frequency of the SHIP-TREND imputation**

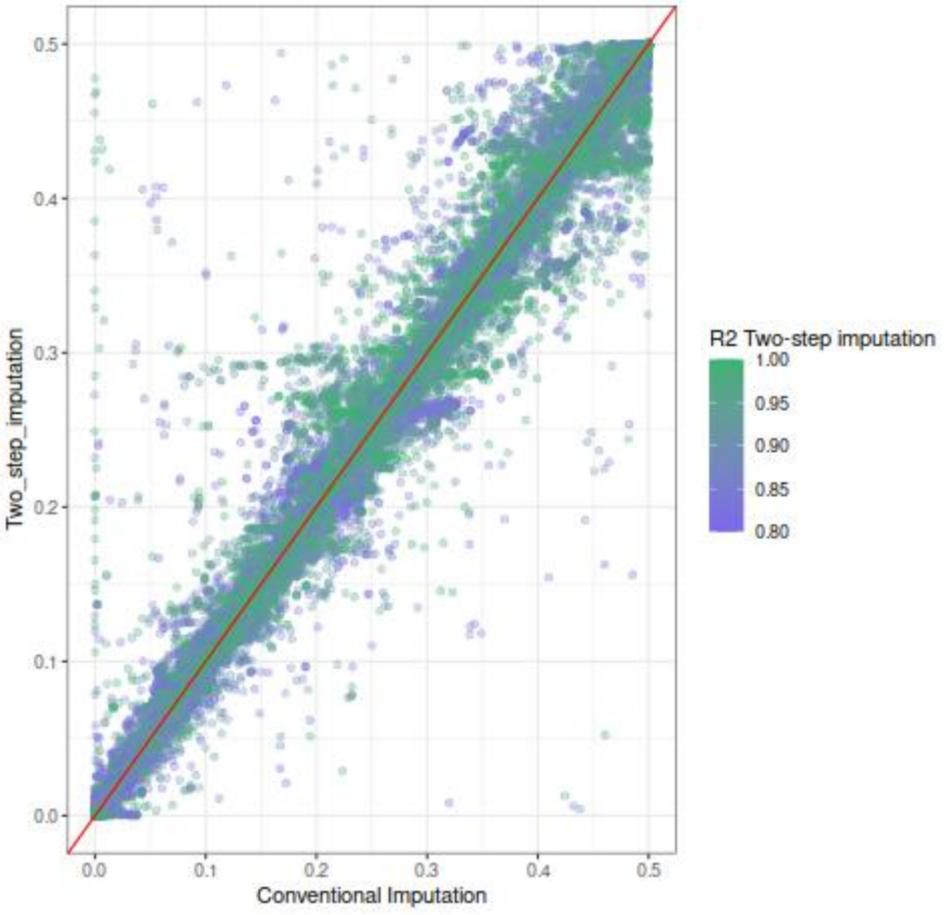


Scatter plot of the allele frequency between conventional (x axis) and two-step imputation (y axis) outcomes coloured by the imputation $R^2$ of the two-step imputation. Variants were selected by a minimum $R^2 = 0.8$.

**Supplementary Figure 13. Manhattan plot of the GWAS analysis of goiter risk using combined two-step imputation genotypes meta-analysed by METAL.**

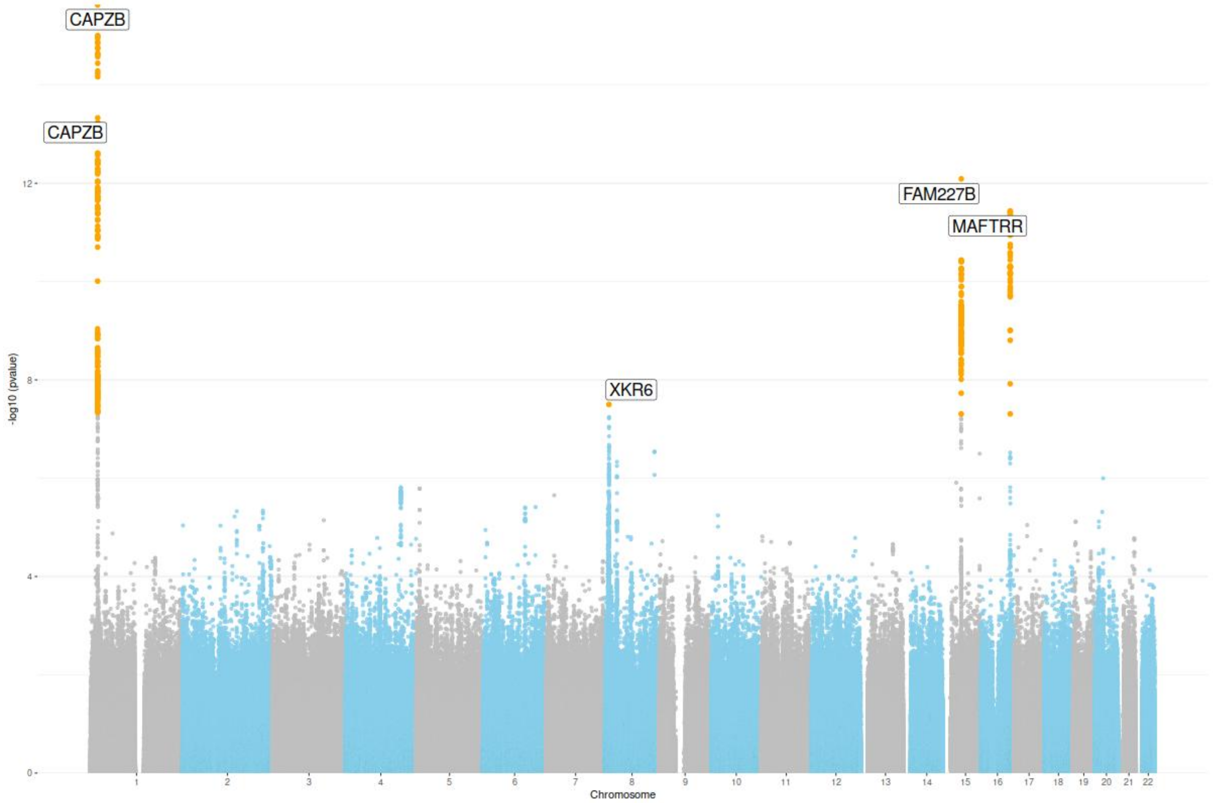


Variants are plotted on the x axis and $-\log_{10}$ p-values of the association testing on the y axis. Associations significant after correction for multiple testing ($p < 5 \times 10^{-8}$) are colored in yellow.

## Supplementary Tables

**Supplementary Table 1. Distribution of the included variants for imputation against 1000G reference panel in the proposed two-step imputation with an $R^2$ threshold of 0.8 and 0.9 respectively.**

| chromosome | $R^2$ threshold = 0.8 | $R^2$ threshold = 0.9 | Overlap 5 cohorts | Overlap 3 cohorts |
|---|---|---|---|---|
| 1 | 161668 | 146001 | 935 | 4756 |
| 2 | 174018 | 155530 | 958 | 4684 |
| 3 | 146475 | 130690 | 904 | 3926 |
| 4 | 135205 | 121440 | 728 | 3387 |
| 5 | 130083 | 115862 | 728 | 3492 |
| 6 | 141011 | 124891 | 976 | 4025 |
| 7 | 115696 | 103837 | 736 | 3217 |
| 8 | 114410 | 101695 | 633 | 3003 |
| 9 | 94958 | 85149 | 569 | 2666 |
| 10 | 108586 | 97452 | 624 | 3121 |
| 11 | 104556 | 93927 | 566 | 2981 |
| 12 | 100130 | 89775 | 656 | 2909 |
| 13 | 76257 | 67913 | 501 | 2196 |
| 14 | 68389 | 62085 | 432 | 1940 |
| 15 | 63205 | 57674 | 383 | 1912 |
| 16 | 67940 | 62968 | 402 | 1970 |
| 17 | 56561 | 53446 | 338 | 1669 |
| 18 | 60575 | 55305 | 386 | 1797 |
| 19 | 42058 | 40105 | 227 | 1110 |
| 20 | 50755 | 46494 | 304 | 1617 |
| 21 | 28374 | 26104 | 175 | 879 |
| 22 | 30017 | 28608 | 104 | 834 |
| Total | 2070927 | 1866951 | 12265 | 58091 |

The last two columns show the distribution of the overlapped variants in all five cohorts and in only the three SHIP cohorts, respectively.

**Supplementary Table 2. Differences in median $R^2$ between conventional and two-step imputation approaches**

| Cohort | AF category | Median $R^2$ (conventional) | Median $R^2$ (Two-step) | Median AF difference |
|---|---|---|---|---|
| GANI_MED | 0-0.001 | 0.01392 | 0.01094 | 0.00008 |
| | 0.001-0.01 | 0.2227 | 0.56329 | 0.00098 |
| | 0.01-0.02 | 0.63723 | 0.905425 | 0.00207 |
| | 0.02-0.03 | 0.71919 | 0.96703 | 0.00253 |
| | 0.03-0.04 | 0.76575 | 0.98345 | 0.00279 |
| | 0.04-0.05 | 0.79908 | 0.98827 | 0.00303 |
| | 0.05-0.5 | 0.90394 | 0.99093 | 0.00695 |
| SHIP-START | 0-0.001 | 0.02052 | 0.01386 | 0.00004 |
| | 0.001-0.01 | 0.4101 | 0.57362 | 0.00067 |
| | 0.01-0.02 | 0.7702 | 0.90913 | 0.00121 |
| | 0.02-0.03 | 0.86806 | 0.96962 | 0.0013 |
| | 0.03-0.04 | 0.92255 | 0.98555 | 0.00128 |
| | 0.04-0.05 | 0.94684 | 0.98974 | 0.00128 |
| | 0.05-0.5 | 0.97457 | 0.9921 | 0.00207 |
| SHIP-START II | 0-0.001 | 0.00076 | 0.00016 | 0.00004 |
| | 0.001-0.01 | 0.061155 | 0.10509 | 0.00172 |
| | 0.01-0.02 | 0.64082 | 0.89676 | 0.00465 |
| | 0.02-0.03 | 0.787305 | 0.96166 | 0.005 |
| | 0.03-0.04 | 0.86594 | 0.98326 | 0.00495 |
| | 0.04-0.05 | 0.90454 | 0.98996 | 0.00494 |
| | 0.05-0.5 | 0.94719 | 0.98935 | 0.00822 |
| SHIP-TREND | 0-0.001 | 0.0033 | 0.00373 | 0 |
| | 0.001-0.01 | 0.63978 | 0.63666 | 0.00016 |
| | 0.01-0.02 | 0.91488 | 0.91633 | 0.00017 |
| | 0.02-0.03 | 0.97447 | 0.97491 | 0.00012 |
| | 0.03-0.04 | 0.98762 | 0.98785 | 0.0001 |
| | 0.04-0.05 | 0.99217 | 0.99227 | 0.00008 |
| | 0.05-0.5 | 0.993 | 0.99336 | 0.00017 |
| SHIP-TREND B2 | 0-0.001 | 0.01563 | 0.01239 | 0.00006 |
| | 0.001-0.01 | 0.37792 | 0.57986 | 0.00078 |
| | 0.01-0.02 | 0.81587 | 0.91021 | 0.00124 |
| | 0.02-0.03 | 0.90292 | 0.96922 | 0.00132 |
| | 0.03-0.04 | 0.92814 | 0.98479 | 0.00139 |
| | 0.04-0.05 | 0.93044 | 0.9892 | 0.00154 |
| | 0.05-0.5 | 0.93135 | 0.99131 | 0.00443 |

Results of the included five cohorts are stratified by allele frequency (AF).

**Supplementary Table 3. Genotype concordance with sequenced data stratified by allele frequency.**

| Group | All variants | AF 0.05-1 | AF 0.01-0.05 | AF 0.0-0.01 |
|---|---|---|---|---|
| **HomRef (n)** | 0.9962 (613,997,680) | 0.9910 (222,769,137) | 0.9988 (183,771,150) | 0.9994 (207,703,823) |
| **Het (n)** | 0.9810 (116,144,961) | 0.9893 (105,617,755) | 0.9418 (8,903,479) | 0.7525 (1,695,994) |
| **HomAlt (n)** | 0.9787 (21,408,323) | 0.9807 (21,290,637) | 0.7862 (124,359) | 0.1389 (2,369) |

Genotype concordance (percentage of the number of matching genotypes/total number of genotypes) of the hard call of the two-step imputed genotypes with sequenced data for homozygous reference (HomRef), homozygous alternative (HomAlt), and heterozygous (Het) calls for all variants and stratified by allele frequency (AF). (n) represents number of variants represented per group in both imputed and sequenced genotypes.

**Supplementary Table 4. Genotype concordance with sequenced data stratified by imputation quality $R^2$.**

| Group | All variants | | $R^2 \geq 0.7$ | | $0.7 > R^2 \geq 0.3$ | | $R^2 < 0.3$ | |
|---|---|---|---|---|---|---|---|---|
| | Conventional | Two-step | Conventional | Two-step | Conventional | Two-step | Conventional | Two-step |
| **HomRef** | 0.9965 | 0.9962 | 0.9963 | 0.9962 | 0.9952 | 0.995 | 0.9922 | 0.9915 |
| **Het** | 0.982 | 0.981 | 0.987 | 0.9866 | 0.6388 | 0.6347 | 0.9231 | 0.923 |
| **HomAlt** | 0.9785 | 0.9787 | 0.9815 | 0.9818 | 0.5423 | 0.5434 | 0.9333 | 0.936 |

Genotype concordance (percentage of the number of matching genotypes/total number of genotypes) of the hard call imputed genotypes with sequenced data for homozygous reference (HomRef), homozygous alternative (HomAlt), and heterozygous (Het) calls for all variants and stratified by imputation quality $R^2$.

## References

Grabe,H.J. *et al.* (2014) Cohort profile: Greifswald approach to individualized medicine (GANI_MED). *J. Transl. Med.*, **12**, 144.

Kang HM (2016) EPACTS: Efficient and Parallelizable Association Container Toolbox.

Völzke,H. *et al.* (2011) Cohort Profile: The Study of Health in Pomerania. *Int. J. Epidemiol.*, **40**, 294–307.
